# Serum circulating proteins from pediatric patients with dilated cardiomyopathy cause pathologic remodeling and cardiomyocyte stiffness

**DOI:** 10.1172/jci.insight.148637

**Published:** 2021-10-08

**Authors:** Danielle A. Jeffrey, Julie Pires Da Silva, Anastacia M. Garcia, Xuan Jiang, Anis Karimpour-Fard, Lee S. Toni, Thomas Lanzicher, Brisa Peña, Carissa A. Miyano, Karin Nunley, Armin Korst, Orfeo Sbaizero, Matthew R.G. Taylor, Shelley D. Miyamoto, Brian L. Stauffer, Carmen C. Sucharov

**Affiliations:** 1 Department of Medicine and; 2 Department of Pediatrics, Division of Cardiology, University of Colorado Anschutz Medical Campus, Children’s Hospital Colorado, Aurora, Colorado, USA.; 3 Department of Pharmacology, University of Colorado Anschutz Medical Campus, Aurora, Colorado, USA.; 4 Department of Engineering and Architecture, University of Trieste, Trieste, Italy.; 5 Bioengineering Department, University of Colorado Denver Anschutz Medical Campus, Aurora, Colorado, USA.; 6 Department of Medicine, Division of Cardiology, Denver Health Medical Center, Denver, Colorado, USA.

**Keywords:** Cardiology, Cardiovascular disease, Molecular biology

## Abstract

Dilated cardiomyopathy (DCM) is the most common form of cardiomyopathy and main indication for heart transplantation in children. Therapies specific to pediatric DCM remain limited due to lack of a disease model. Our previous study showed that treatment of neonatal rat ventricular myocytes (NRVMs) with serum from nonfailing or DCM pediatric patients activates the fetal gene program (FGP). Here we show that serum treatment with proteinase K prevents activation of the FGP, whereas RNase treatment exacerbates it, suggesting that circulating proteins, but not circulating miRNAs, promote these pathological changes. Evaluation of the protein secretome showed that midkine (MDK) is upregulated in DCM serum, and NRVM treatment with MDK activates the FGP. Changes in gene expression in serum-treated NRVMs, evaluated by next-generation RNA-Seq, indicated extracellular matrix remodeling and focal adhesion pathways were upregulated in pediatric DCM serum and in DCM serum–treated NRVMs, suggesting alterations in cellular stiffness. Cellular stiffness was evaluated by Atomic Force Microscopy, which showed an increase in stiffness in DCM serum–treated NRVMs. Of the proteins increased in DCM sera, secreted frizzled-related protein 1 (sFRP1) was a potential candidate for the increase in cellular stiffness, and sFRP1 treatment of NRVMs recapitulated the increase in cellular stiffness observed in response to DCM serum treatment. Our results show that serum circulating proteins promoted pathological changes in gene expression and cellular stiffness, and circulating miRNAs were protective against pathological changes.

## Graphical abstract

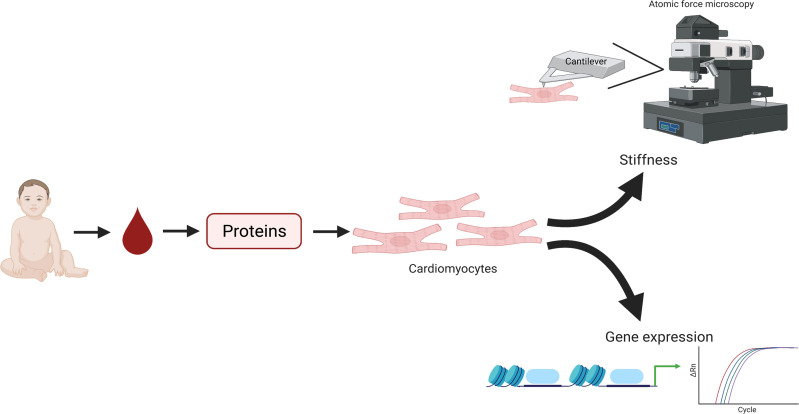

## Introduction

Heart failure (HF) is a major public health issue in the United States and a leading cause of hospitalization in both adult and pediatric populations (1). In children, HF has several etiologies, with congenital heart disease being the most common reason for heart transplantation in children younger than 1 year of age, and dilated cardiomyopathy (DCM) being the most prevalent reason in children older than 1 year of age (2). Within 5 years of diagnosis, 40% of children with DCM either die or undergo cardiac transplantation (3). Although DCM is present in both adult and pediatric populations, we and others have identified age-specific differences, including changes in β-adrenergic receptor (β-AR) density, a unique transcriptome profile, lack of cardiomyocyte hypertrophy, and minimal interstitial fibrosis in the pediatric population when compared with adults (4–7). Although there is minimal fibrosis, extracellular matrix (ECM) remodeling is present in the pediatric DCM heart (5, 8). Alterations in ECM may increase myocardial stiffness, which could lead to ventricular dysfunction (9). Although stiffness in adults with HF has been extensively studied, less is known about the contribution of stiffness to HF in the pediatric population (10).

One major limitation to pediatric studies is the lack of an in vivo disease model. To circumvent this problem, we developed an in vitro model that consists of treating primary cardiomyocytes with serum from pediatric patients with DCM. We previously showed that serum circulating factors contribute to pathologic remodeling in vitro using neonatal rat ventricular myocytes (NRVMs) and human-induced pluripotent stem cell–derived cardiomyocytes (4, 11). Additionally, we showed that serum-induced pathological remodeling was not dependent on the β-AR or the renin-angiotensin-aldosterone systems, and that factors in the secretome, other than circulating catecholamines or angiotensin II, contribute to pathological cellular remodeling (11).

The secretome contains proteins, DNA, and small noncoding RNAs. Often these factors are present in extracellular vesicles (EVs). Our previous work showed that exosomes/EVs are involved in the pathological response to DCM serum (11). EVs can function as paracrine factors and can carry proteins and nucleic acids, including miRNAs. miRNAs are small noncoding RNAs, roughly 22 nucleotides in length, that are capable of modulating gene expression by interacting with the 3′-UTR of target mRNAs (12). EV miRNAs can alter gene expression in recipient cells as shown in various cancer models, whereas miRNAs modulate processes such as angiogenesis, drug resistance, and metastasis (13, 14). Additionally, others have shown that paracrine EV miRNA can contribute to cardiomyocyte hypertrophy through cell-to-cell communication between cardiac fibroblasts and cardiomyocytes (15). Although we previously showed that EVs, including exosomes, and the resulting serum suspension (EV-free fraction) can contribute to pathologic remodeling in NRVMs, the number of exosomes in pediatric DCM serum was lower than that in nonfailing (NF) controls (11). Furthermore, we did not investigate which serum secretome factors were important for the observed pathologic response. The goal of this study was to define the contribution of circulating miRNAs and proteins, regardless of EV localization, to pathologic remodeling in cardiomyocytes, and the functional consequence of DCM serum treatment in vitro.

Using a multiomics approach, we sought to define changes in gene expression in response to DCM serum treatment of NRVMs compared with cells treated with age-matched NF control serum. Here we show that the pathways related to ECM remodeling and focal adhesion were upregulated in the transcriptome of DCM-treated primary cardiomyocytes. Furthermore, we show that changes in the expression of the fetal gene program (FGP) were mediated by serum circulating proteins, that serum circulating miRNAs were protective, and that, of the significantly altered miRNAs, all were decreased in pediatric DCM serum when compared with serum from NF controls. An analysis of the circulating proteome profile of children with DCM identified several upregulated proteins. Our results show that the treatment of NRVMs with recombinant midkine (MDK) increased the expression of atrial natriuretic factor (ANF) and b-type natriuretic peptide (BNP), and serum depletion of MDK blunted serum-mediated increases in ANF and BNP. To determine the functional significance of the predicted alterations in ECM remodeling and focal adhesion, we performed atomic force microscopy (AFM) of serum-treated NRVMs. Our results show an increase in stiffness in DCM serum–treated cells. Of the proteins increased in the DCM secretome, secreted frizzled-related protein 1 (sFRP1) was a likely contributor to the observed stiffness, because the treatment of NRVMs with recombinant sFRP1 resulted in increased stiffness. In summary, we provide evidence to support the secretome as an important contributor to pathologic cardiomyocyte remodeling, and the use of serum-treated NRVMs as a valid model to investigate pediatric DCM-related mechanisms of disease.

## Results

### Patient characteristics.

Summarized patient characteristics for all patients included in this study are listed in Table 1 and detailed characteristics in Supplemental Table 1 (supplemental material available online with this article; https://doi.org/10.1172/jci.insight.148637DS1). NF patient serum and plasma had a total number of 23 subjects, with a median age of 9.74 (IQR range of 6.19), 39% of whom were female. DCM patient serum and plasma had a total number of 49 subjects, with a median age of 4.83 (IQR range of 11.09), 52% of whom were female.

### ECM remodeling pathways were altered in response to DCM serum–treated NRVMs.

**Figure 1:**
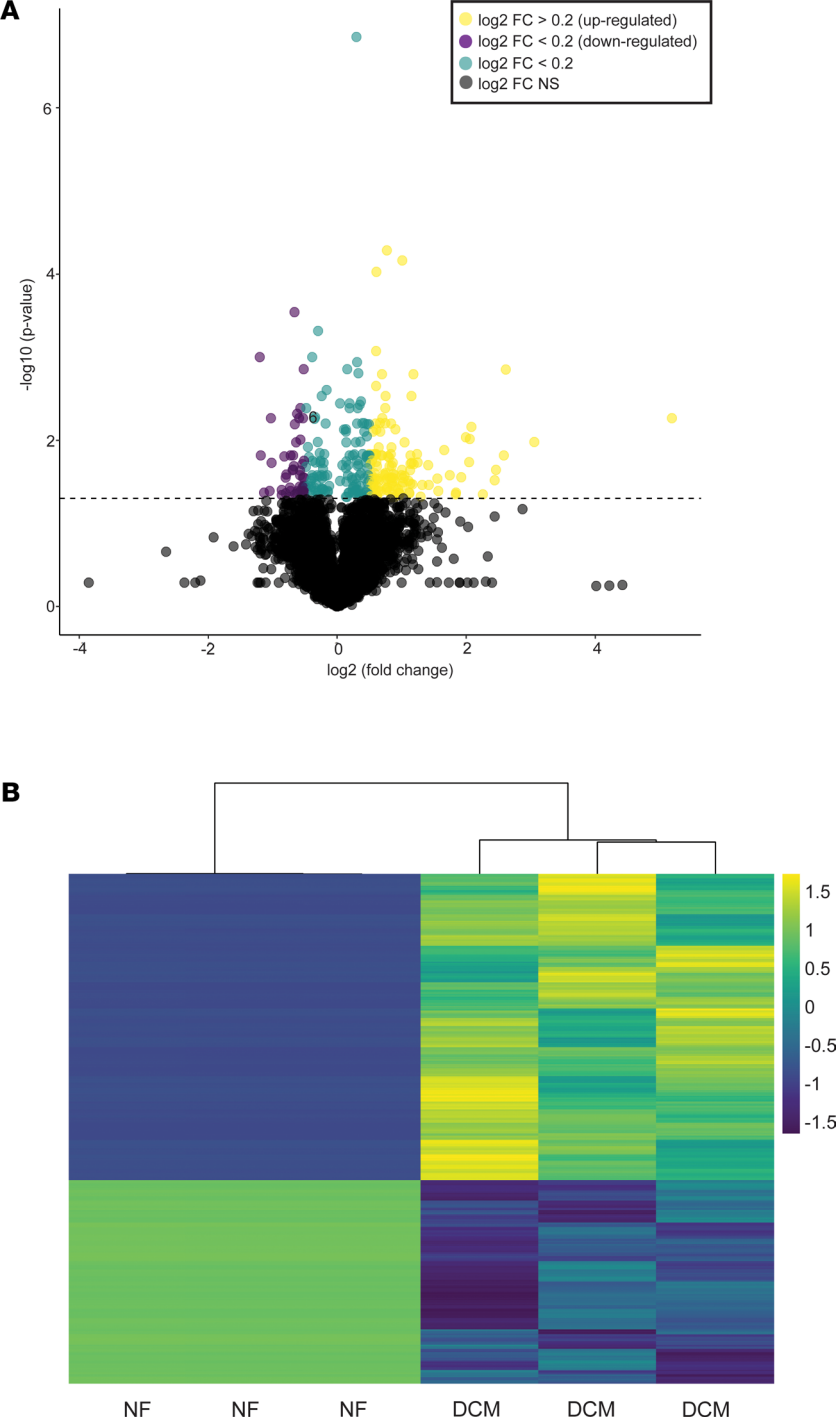
DCM serum elicits significant transcriptional changes in NRVMs. (**A**) Volcano plot representation of RNA-Seq transcripts significantly differentially expressed (4079 genes total) in NRVMs treated with NF (*n* = 6) and DCM (*n* = 6) serum from 3 independent NRVM preps. Log2 fold change is represented on the *x* axis and –log10 *q* value is represented on the *y* axis. Horizontal dashed line represents significance cutoff, Welch’s 2-tailed *t* test, assuming equal variance *q* < 0.10. (**B**) Heatmap representing unsupervised clustering of NF serum–treated (*n* = 6) and DCM serum–treated (*n* = 6) NRVMs with 629 significantly expressed transcripts. DCM, dilated cardiomyopathy; NRVMs, neonatal rat ventricular myocytes; NF, nonfailing, FC, fold change.

To further explore pathological remodeling associated with pediatric DCM, we evaluated gene expression changes in NF serum– and DCM serum–treated NRVMs. Next-generation RNA-Seq was performed on serum-treated NRVMs (*n* = 6 NF serum–treated and *n* = 6 DCM serum–treated NRVMs from 3 different NRVM preparations) and identified more than 20,000 genes. We identified 629 significantly differentially expressed genes between NF serum– and DCM serum–treated samples, 378 upregulated and 251 downregulated (Figure 1A). Unsupervised hierarchical clustering separated NF and DCM samples based on their gene expression profiles (Figure 1B). Supplemental Table 2 lists all significantly 629 differentially expressed genes.

Using IPA and Metascape, pathways were analyzed using the identified significantly differentially expressed genes (Table 2). The top 15 canonical pathways of both upregulated (312 mapped IDs) and downregulated (227 mapped IDs) genes are reported. ECM organization, including collagen formation, angiogenesis, and focal adhesion, were substantially upregulated pathways. Canonical pathways related to downregulated genes included metabolism of carbohydrates, NOD-like receptor signaling pathway, positive regulation of cardiac muscle hypertrophy, and protein destabilization. Using IPA, top differentially regulated cardiotoxicity functions identified cardiac enlargement, heart failure, and cardiac cell death with cardiac dysfunction (Table 3).

### DNase and RNase affected fetal gene program response.

To determine if circulating DNA or RNA present in serum could elicit pathological changes in gene expression, NF and DCM sera were subjected to heat/freeze to expose EVs or protein-bound nucleic acid contents (16), and treated with vehicle, DNase (NF *n* = 4; DCM *n* = 8) from 7 independent experiments, or RNase (NF *n* = 5; DCM *n* = 9) from 9 independent experiments. To ensure these nucleic acids were taken up by cells in the absence of EVs, the resulting serum products were transfected into NRVMs.

**Figure 2:**
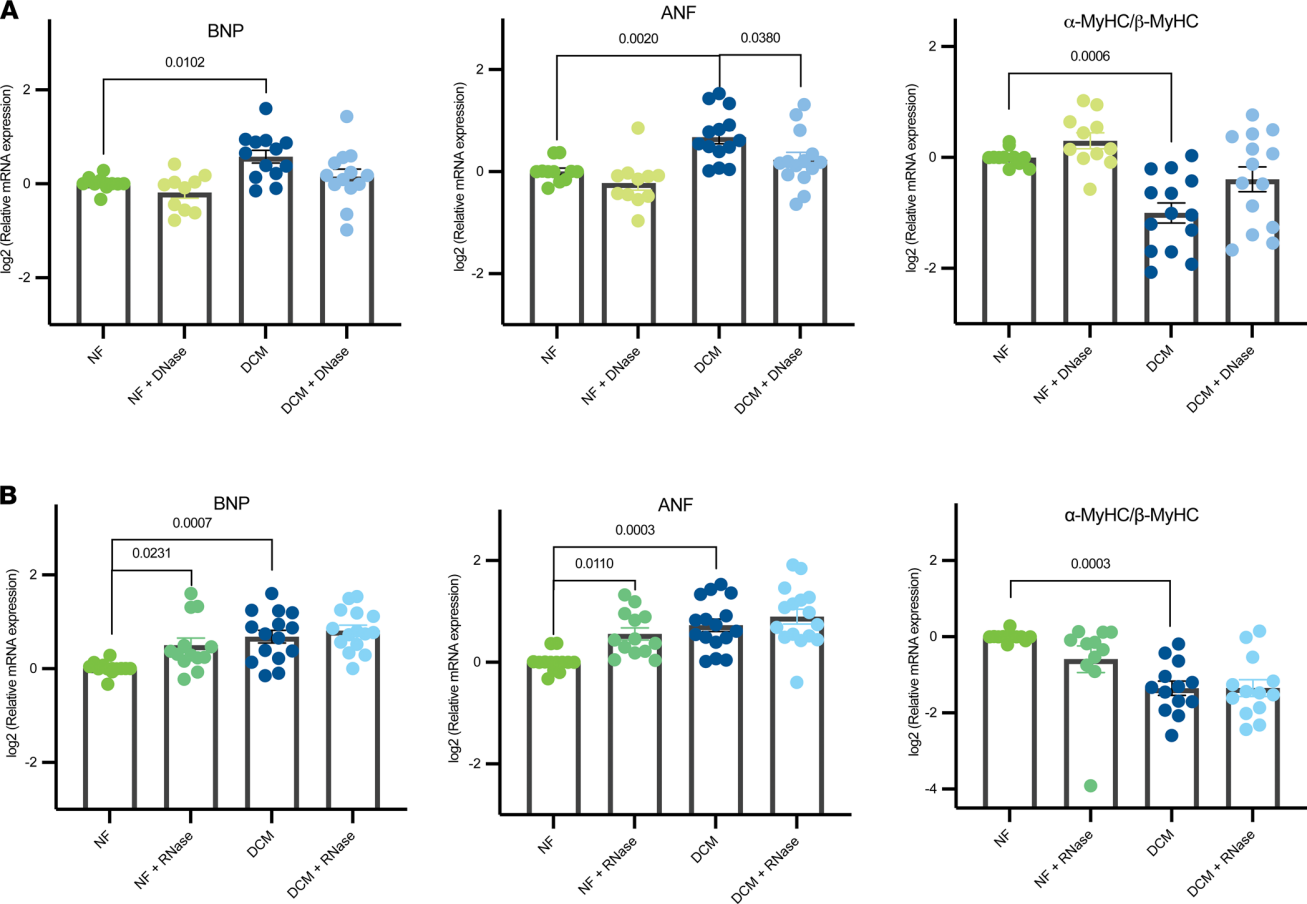
Fetal gene program expression changes in DCM serum– and NF serum–treated NRVMs with or without DNase and RNase. (**A**) RT-qPCR of FGP expression in NRVMs transfected with serum with or without DNase. Expression of BNP, ANF, and α-MyHC to β-MyHC ratios. Gene expression was normalized to 18S, and data are presented as a relative fold change to NF controls. DCM *n* = 8, NF *n* = 4 from 7 independent NRVM preps. (**B**) RT-qPCR of FGP expression analysis of serum-treated NVRMs with or without RNase. Expression of BNP, ANF, and α-MyHC/β-MyHC was evaluated. Gene expression was normalized to 18S, and data are presented as a relative fold change to NF controls. DCM *n* = 9, NF *n* = 5 from 9 independent NRVM preps. All groups are log2 transformed and error bar denotes mean ± SEM. *P* values are notated in the figure. Fitting a mixed model, Sidak’s multiple comparisons test was used for all data sets. DCM, dilated cardiomyopathy; NRVMs, neonatal rat ventricular myocytes; NF, nonfailing; FGP, fetal gene program; BNP, natriuretic peptide B; ANF, atrial natriuretic factor; α-MyHC, α-myosin heavy chain; β-MyHC, β-myosin heavy chain.

Changes in cardiac gene expression, commonly known as the fetal gene program (FGP), are a hallmark of pathologic remodeling and characterized by the reexpression of embryonically expressed genes and repression of adult genes (17). NRVMs treated with DCM patient sera display recapitulation of the FGP, including the upregulation of BNP, ANF, and a significantly decreased ratio of α-myosin heavy chain (α-MyHC) to β-myosin heavy chain (β-MyHC; Figure 2). NF DNase–treated NRVMs showed no significant changes in the FGP compared with untreated controls (Figure 2A). When DCM serum was treated with DNase, there were no significant changes in the expression of BNP or α-MyHC/β-MyHC ratio; however, there was a significant decrease in the expression of ANF (*P* = 0.038). In contrast, RNase treatment of DCM serum did not affect changes in gene expression in response to serum treatment. However, RNase-treated NF serum showed an increase in BNP (*P* = 0.023) and ANF (*P* = 0.011), with no significant changes in α-MyHC/β-MyHC ratios (Figure 2B).

### Circulating miRNAs from DCM serum were primarily downregulated and were related to canonical pathways involved in remodeling and upregulated cardiotoxic pathways.

**Figure 3:**
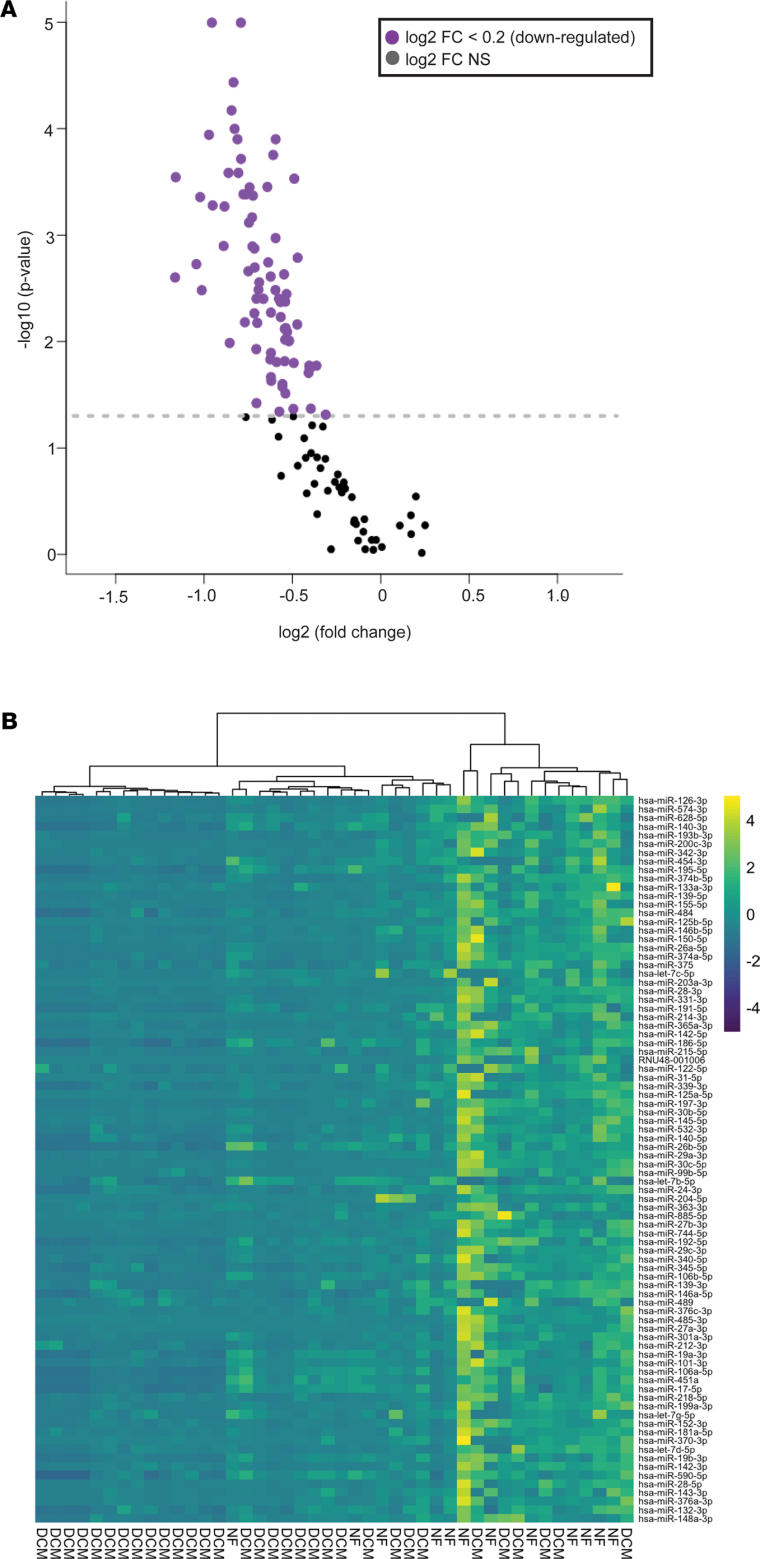
Circulating miRNAs are dysregulated in serum from pediatric patients with DCM. (**A**) Volcano plot representation of the 123 differentially expressed miRNAs between NF (*n* = 12) and DCM (*n* = 32) patients. Log2 fold change is represented on the *x* axis and –log10 *q* value differentially expressed on the *y* axis. Horizontal line indicates significance; Wilcoxon’s test *q* < 0.10. (**B**) Heatmap of the 84 significantly differentially expressed circulating miRNAs. Unsupervised hierarchical clustering separated NF (*n* = 12) and DCM (*n* = 32) patients; Wilcoxon’s test *q* < 0.10. DCM, dilated cardiomyopathy; NF, nonfailing; FC, fold change.

miRNA array analysis of serum from NF (*n* = 12) and DCM (*n* = 32) patients identified 123 total miRNAs; 7 miRNAs were upregulated and 116 miRNAs were downregulated. All 84 significantly differentially regulated miRNAs were downregulated in DCM serum (Figure 3A and Supplemental Table 3). Unsupervised hierarchical clustering showed most samples clustered according to phenotype (NF or DCM), as represented by the heatmap (Figure 3B).

Because IPA and Metascape do not predict pathways based solely on miRNA expression, to investigate pathways that may be affected by alterations in circulating miRNAs, pathway analysis was conducted using miRPathDB (18). To minimize false-positive pathways, we restricted the analysis to a minimum of 30 miRNAs (or one-third of the significantly dysregulated miRNAs) required to target any 1 pathway (Supplemental Table 5). We observed predicted dysregulation of pathways related to cancer and viral infection, suggesting alterations in cell cycle and inflammatory pathways. Interestingly, focal adhesion signaling and PI3k/AKT signaling pathway were enriched in the pathway analysis. IPA analysis of the 84 significantly dysregulated miRNAs indicated multiple cardiotoxicity pathways, including cardiac enlargement, cardiac dilation, and cardiac fibrosis (Table 4).

### Proteinase K treatment of DCM serum prevented activation of the FGP.

**Figure 4:**
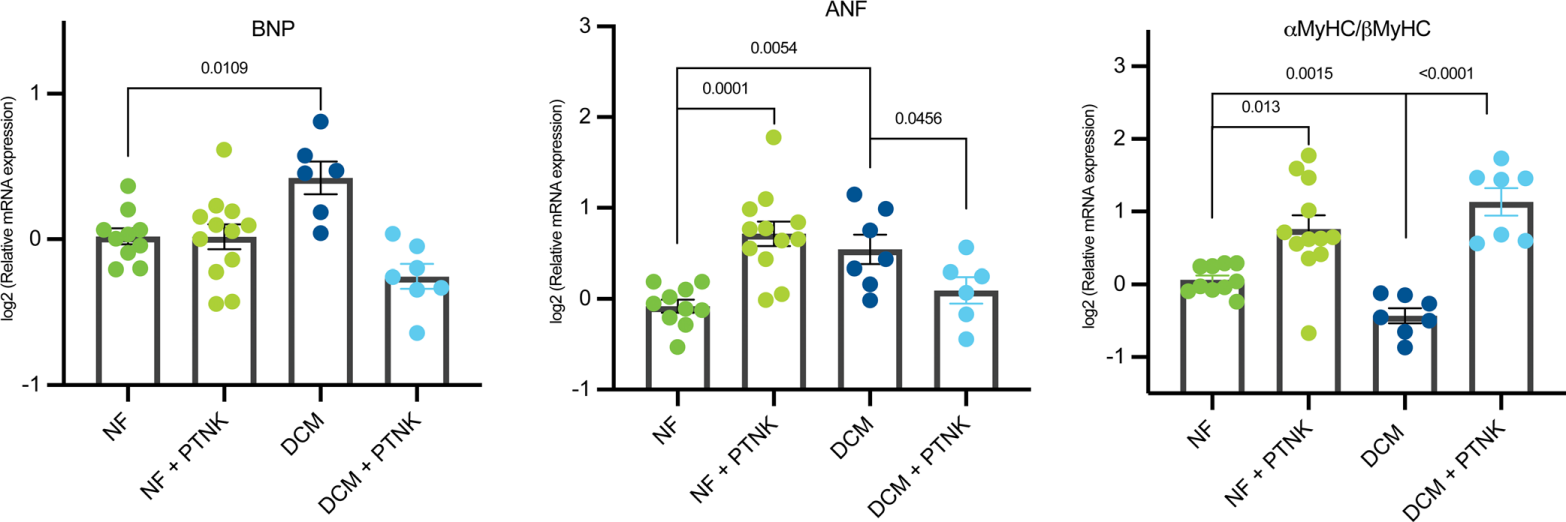
DCM serum activation of the FGP is mediated by circulating proteins. RT-qPCR of FGP expression analysis of serum-treated NVRMs with or without PTNK. Expression of BNP, ANF, and α-MyHC/β-MyHC was evaluated. Gene expression was normalized to 18S. DCM *n* = 4, NF *n* = 4 from 4 different NRVM preps. Data are presented as a relative fold change to NF controls; all groups are log2 transformed and error bar denotes mean ± SEM. *P* values are notated in the figure. Fitting a mixed model, Sidak’s multiple comparisons test was used for all data sets. DCM, dilated cardiomyopathy; NRVMs, neonatal rat ventricular myocytes; NF, nonfailing; FGP, fetal gene program; BNP, natriuretic peptide B; ANF, atrial natriuretic factor; α-MyHC, α-myosin heavy chain; β-MyHC, β-myosin heavy chain; PTNK, proteinase K.

To determine if proteins present in patient sera could induce pathological gene expression changes, NF (*n* = 4) and DCM (*n* = 4) sera were treated with or without proteinase K (PTNK) in 4 independent NRVM experiments (Figure 4). NF sera treated with PTNK showed significant upregulation of ANF (*P* < 0.0001) and α-MyHC/β-MyHC expression (*P* = 0.013). Importantly, PTNK treatment of DCM sera prevented serum-induced pathological gene expression changes with a decrease in BNP (*P* < 0.0001) and ANF (*P* = 0.046) and an increase in the α-MyHC/β-MyHC ratio (*P* < 0.0001).

### Circulating proteins in DCM plasma were involved in activation of remodeling pathways and upregulation of cardiotoxic pathways.

**Figure 5:**
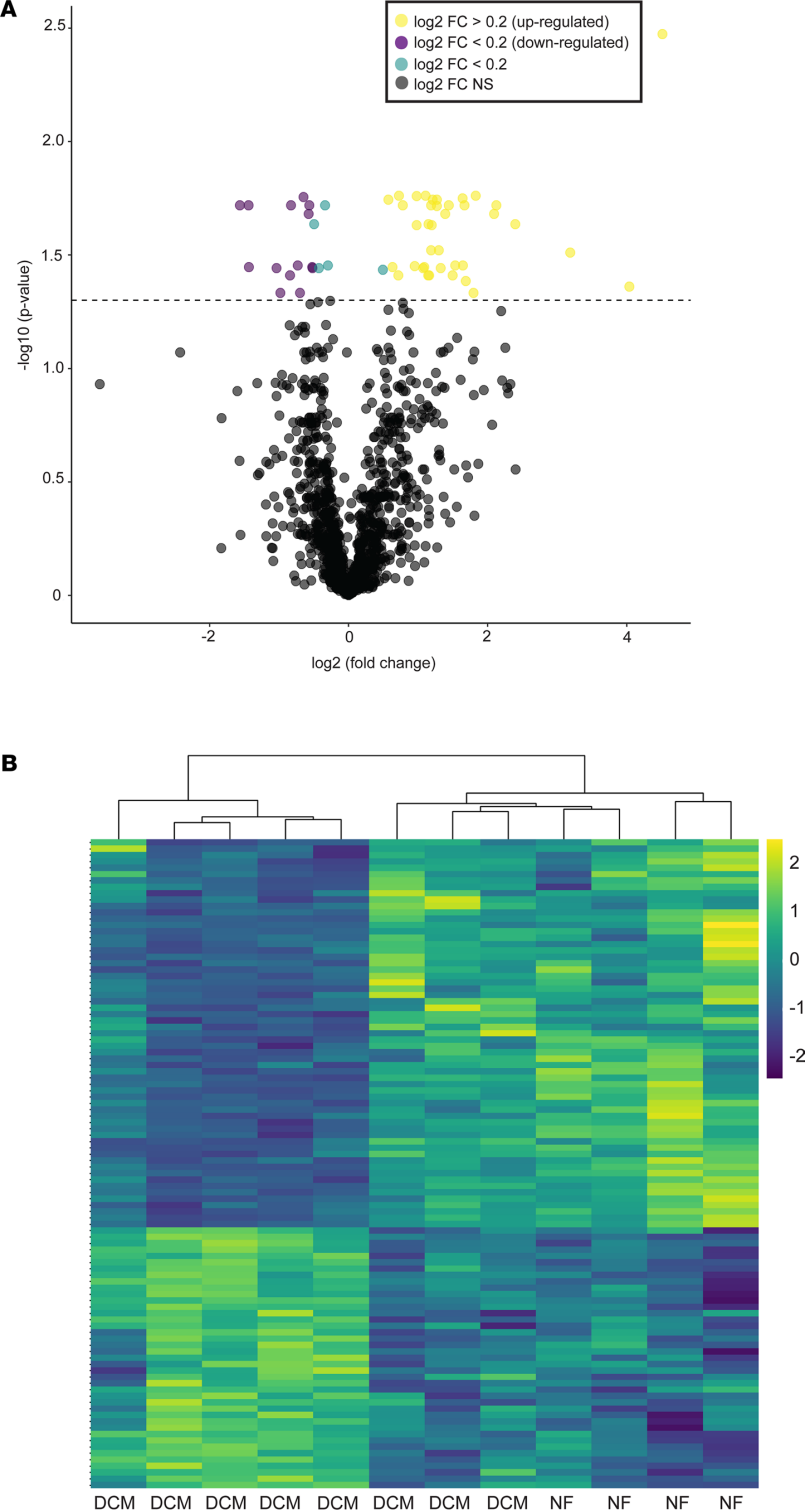
Circulating proteins are dysregulated in plasma of pediatric patients with DCM. (**A**) Volcano plot representation of the 1141 differentially expressed proteins between NF (*n* = 4) and DCM (*n* = 8) patient plasma. Log2 fold change is represented on the *x* axis and –log10 *q* value differentially expressed on the *y* axis. Horizontal dashed line represents significance cutoff; Welch’s *t* test *q* < 0.10. (**B**) Heatmap of the 102 significantly differentially expressed proteins. Unsupervised hierarchical clustering separated NF (*n* = 4) and DCM (*n* = 8) patients; Welch’s *t* test *q* < 0.10. DCM, dilated cardiomyopathy; NF, nonfailing; FC, fold change.

Because our results suggested circulating proteins contributed to pathological remodeling, we conducted SOMAscan-based proteomic analysis of NF (*n* = 4) and DCM (*n* = 8) plasma samples to determine the protein secretome from pediatric patients with DCM. Our analysis detected 1141 proteins, and 102 significantly dysregulated proteins in pediatric DCM plasma were identified (Figure 5, A and B). Of the 102 differentially expressed proteins, 66 proteins were upregulated and 43 were downregulated (Figure 5A). Unsupervised hierarchical clustering using the 102 significantly differentially expressed proteins separated NF and DCM patients (Figure 5B). A list of all 102 significantly differentially expressed proteins is provided in Supplemental Table 4.

Pathway analysis performed using IPA software and Metascape revealed multiple dysregulated canonical pathways associated with DCM patient plasma (Table 5). The top 15 predicted canonical upregulated pathways showed pathways associated with remodeling such as the PI3k/AKT pathway, focal adhesion, and ECM remodeling, along with protein kinase A signaling and positive regulation of cardiac hypertrophy signaling. Pathways associated with decreased protein expression in DCM plasma compared with NF controls included cytokine–cytokine receptor interaction, regulation of leukocyte activation, and apoptosis. A more detailed IPA analysis of cardiac pathways revealed multiple dysregulated cardiotoxicity functions, including heart failure, cardiac enlargement, cardiac necrosis/cell death, and cardiac dysfunction (Table 6).

### Circulating MDK was significantly upregulated in patients with DCM and increased the expression of ANF and BNP.

**Figure 6:**
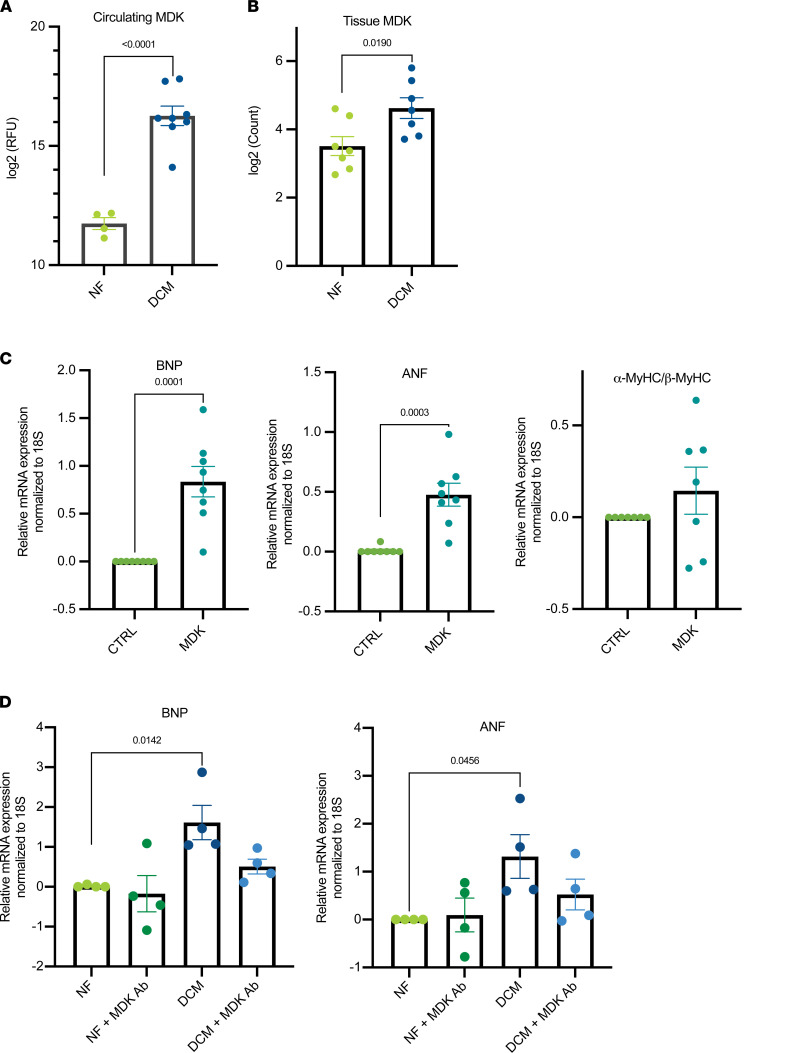
Circulating MDK is upregulated in patients with DCM and increases expression of ANF and BNP. (**A**) Levels of circulating MDK in DCM (*n* = 8) compared with NF plasma (*n* = 4). *P* < 0.0001 by unpaired 2-tailed *t* test. (**B**) Levels of tissue MDK in DCM (*n* = 7) compared with NF (*n* = 7). *P* = 0.019. (**C**) BNP, ANF, and α-MyHC/β-MyHC expression in NRVMs treated with MDK compared with control. *n* = 8 independent experiments. (**D**) DCM serum treatment with MDK antibody blunts the increase in BNP or ANF. *n* = 4 independent experiments. (**C** and **D**) Data are presented as a relative fold change to NF controls; all groups are log2 transformed and error bar denotes mean ± SEM. *P* values are notated in the figure. A comparison between 2 groups was done using unpaired 2-tailed *t* test, whereas a 4-group analysis was done using Sidak’s multiple comparisons test. MDK, midkine; DCM, dilated cardiomyopathy; NRVMs, neonatal rat ventricular myocytes; NF, nonfailing; BNP, natriuretic peptide B; ANF, atrial natriuretic factor; α-MyHC, α-myosin heavy chain; β-MyHC, β-myosin heavy chain.

SOMAscan data indicated the protein MDK was the most upregulated protein in the plasma of pediatric patients with DCM compared with NF controls (Figure 6A). MDK was also increased in the cardiac tissue of pediatric patients with DCM when compared with NF control hearts (based on RNA-Seq data; ref. 4; Figure 6B). To determine if circulating MDK had an effect on pathologic remodeling, NRVMs were treated with human recombinant MDK. As shown in Figure 6C, MDK treatment increased the expression of ANF and BNP, but did not affect the α-MyHC/β-MyHC ratio. Importantly, treatment of serum with the MDK antibody prevented the upregulation of ANF or BNP (Figure 6D).

### Overlapping pathways from the protein secretome and transcriptome from serum-treated NRVMs.

**Figure 7:**
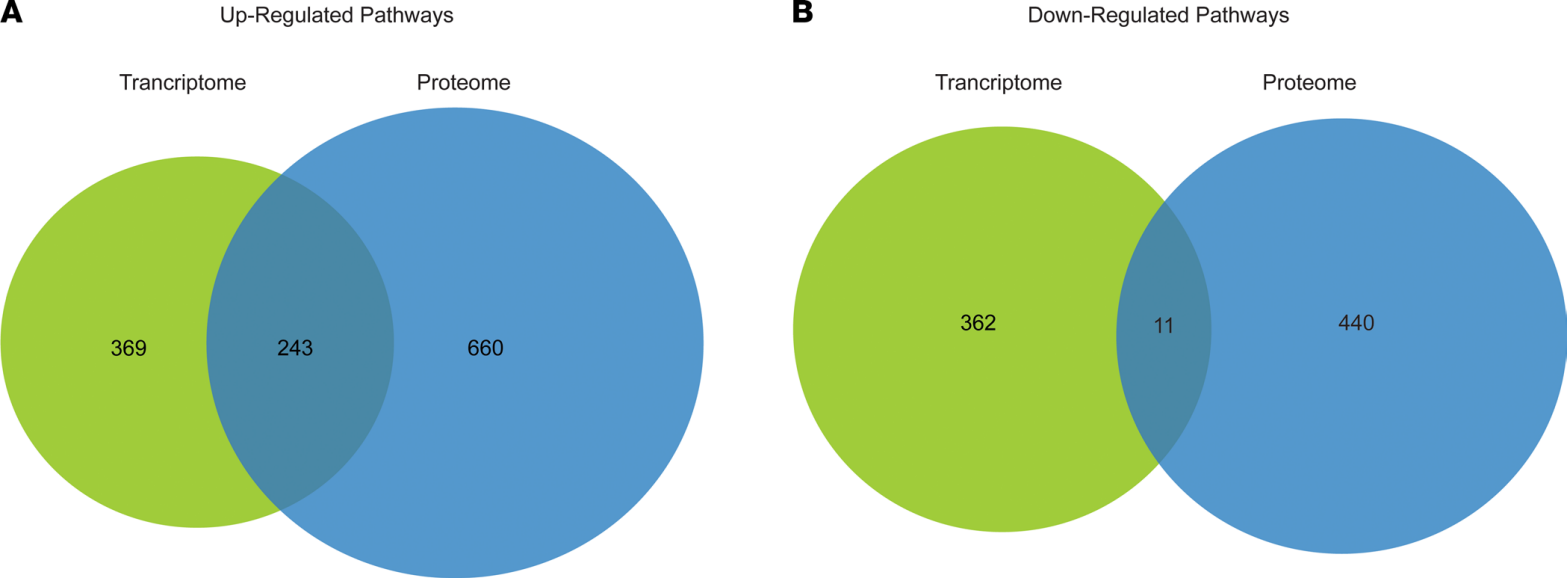
Comparison of upregulated and downregulated from serum-treated NRVM transcripts and circulating proteins found in patient plasma. (**A**) Venn diagram representation of overlapping pathways found to be upregulated based on dysregulated transcripts from DCM serum–treated NRVMs and protein plasma from patients with DCM (369 unique transcriptome pathways, 660 unique protein pathways, and 243 overlapping pathways). (**B**) Venn diagram representation of overlapping pathways found to be downregulated based on dysregulated transcripts from serum-treated NRVMs and protein plasma from patients with DCM (362 unique transcriptome pathways, 440 unique protein pathways, and 11 overlapping pathways). DCM, dilated cardiomyopathy; NRVMs, neonatal rat ventricular myocytes.

To determine if there was an overlap in enriched pathways from the transcriptome of DCM serum–treated NRVMs and differential circulating proteins in DCM plasma, dysregulated pathways generated from Metascape and IPA were compared. Our analysis predicted 369 unique pathways upregulated in NRVMs treated with DCM serum and 660 unique pathways upregulated in the plasma of patients with DCM, of which 243 (23.5%) overlapping pathways are predicted to be commonly upregulated in the NRVM transcriptome and plasma proteome (Figure 7A). Downregulated pathways, however, had fewer overlapping pathways (11, or 1.37%; Figure 7B). The top significant overlapping pathways are shown in Table 7. Focal adhesion is upregulated in both the transcriptome and proteome, further accentuating the importance on this pathway in the DCM patient population.

### Overlapping pathways from DCM serum–treated NRVM and DCM tissue transcriptome suggest alterations in ECM remodeling.

**Figure 8:**
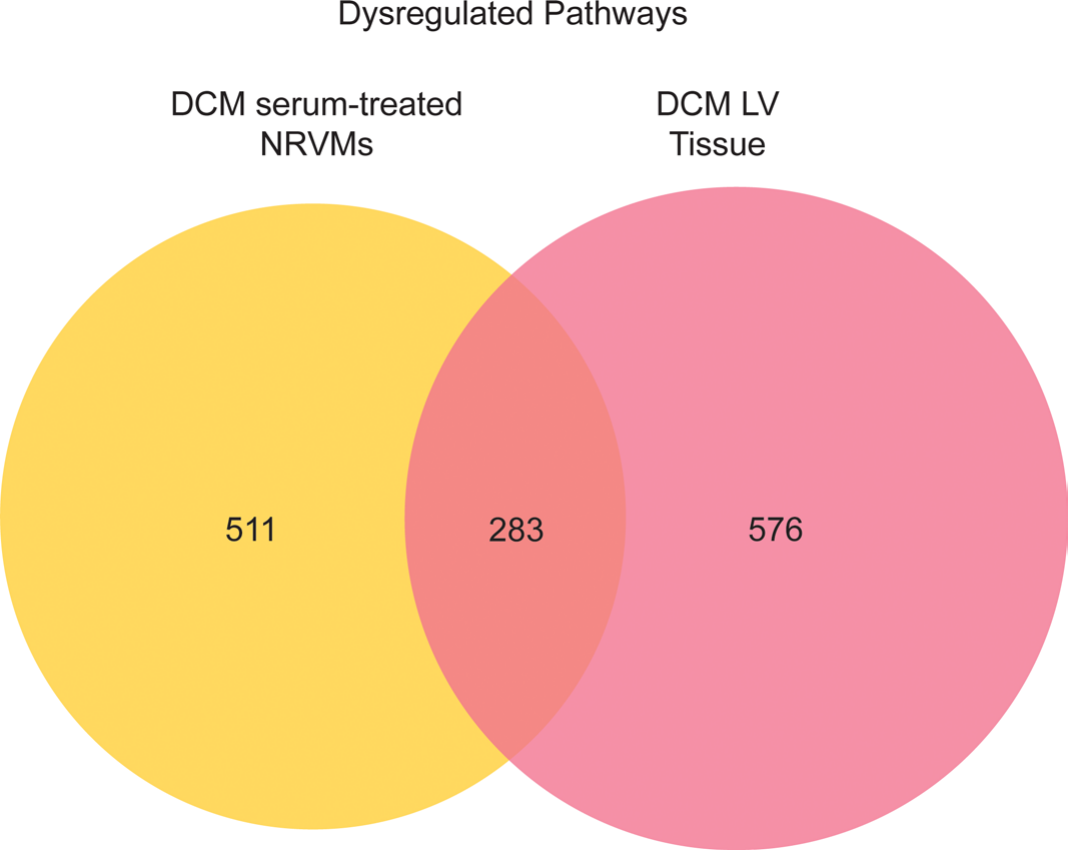
Comparison of pathways commonly altered in the serum-treated NRVM transcriptome and pediatric DCM transcriptome. Venn diagram representation of overlapping pathways altered in pediatric DCM left ventricle (LV) tissue and in serum-treated NRVMs. DCM, dilated cardiomyopathy; NRVMs, neonatal rat ventricular myocytes.

We next evaluated if there was an overlap in the pathways predicted to be altered in NRVMs treated with DCM serum and in pediatric DCM hearts. As shown in Figure 8, using pathway prediction from IPA and Metascape, we identified 283 pathways commonly dysregulated (26.0%) in DCM serum–treated NRVMs and in DCM left ventricle (LV). There were 511 unique dysregulated pathways in DCM serum–treated NRVMs and 576 unique dysregulated pathways in DCM LV tissue (Figure 8). The top overlapping pathways include pathways related to ECM remodeling, signaling by RHO GTPases and collagen formation, biosynthesis, or chain trimerization (Table 8).

### DCM serum treatment promoted stiffness in NRVMs mediated by sFRP1.

**Figure 9:**
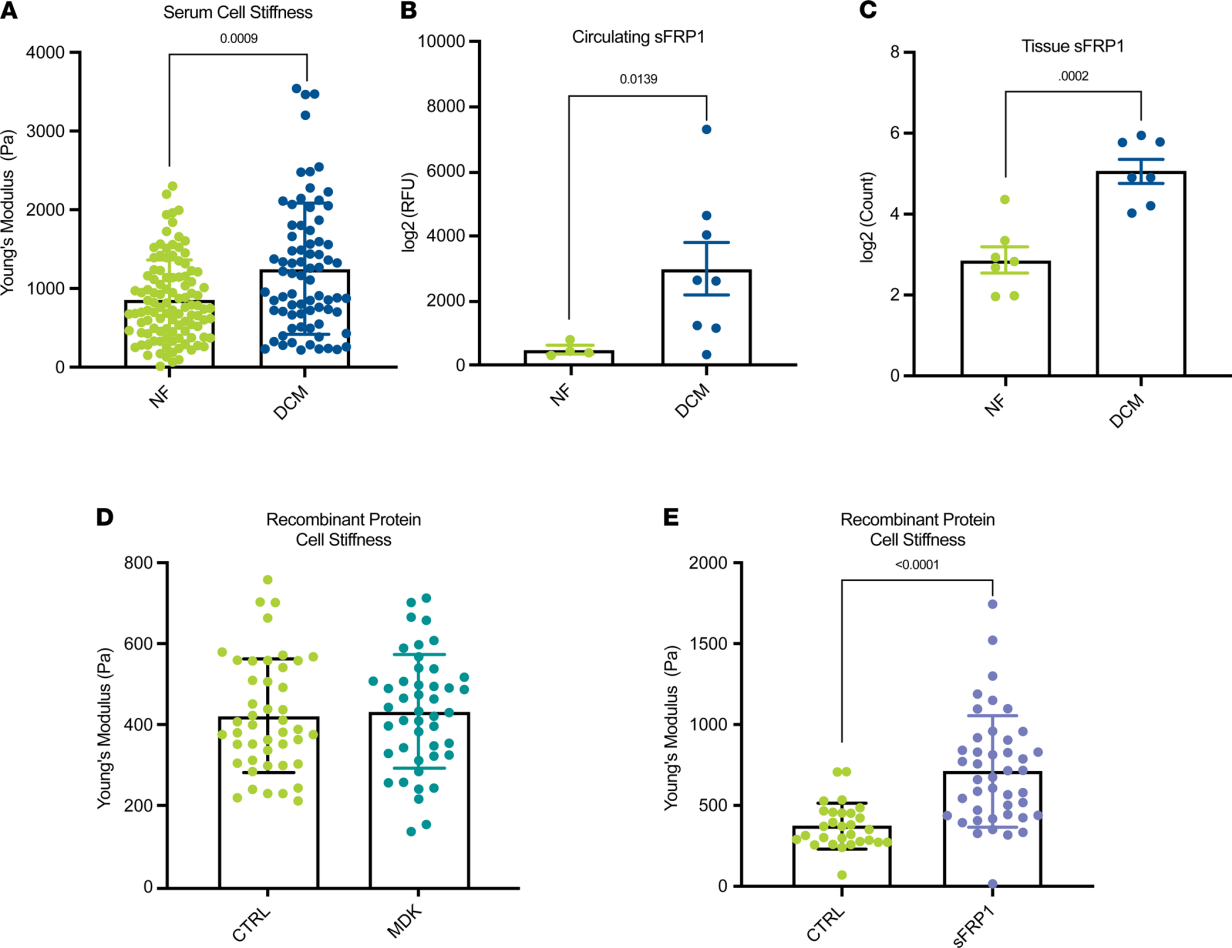
Human recombinant protein sFRP1 increases stiffness in NRVMs. (**A**). Elasticity/stiffness (Young’s modulus) in NRVMs treated with NF and DCM pediatric patient serum. Mann-Whitney *U* test, *P* = 0.0009 (serum). Each dot represents an individual measurement. (**B**) Circulating levels of sFRP1 in DCM (*n* = 8) compared with NF (*n* = 4) controls. *P* = 0.0139. (**C**) Levels of sFRP1 mRNA in pediatric DCM tissue (*n* = 7) compared with NF controls (*n* = 7). *P* = 0.0002. (**D**) NRVMs treated with 1 μg/mL human recombinant MDK for 72 hours. (**E**) NRVMs treated with 1 μg/mL human recombinant (hsFRP1) for 72 hours. *P* < 0.0001. Error bar denotes mean ± SEM. Mann-Whitney *U* *t* test (AFM) or unpaired 2-tailed *t* test (log2 transformed circulating and tissue sFRP1) were used and *P* values are notated in the figure. Mann-Whitney was used in **A**, **D**, and **E** and *t* test in **B** and **C**. DCM, dilated cardiomyopathy; NRVMs, neonatal rat ventricular myocytes; NF, nonfailing; MDK, midkine; AFM, atomic force microscopy; sFRP1, secreted frizzled-related protein 1.

Due to the predicted alterations in ECM remodeling and focal adhesion pathways in response to DCM serum treatment and in the circulating proteomics analysis, we next evaluated cardiomyocyte stiffness by AFM. AFM was performed on NF (*n* = 1) and DCM (*n* = 1) serum–treated NRVMs from 3 individual NRVM preps. DCM-treated NRVMs resulted in a significantly increased Young’s modulus (kPa), a readout of cellular stiffness (Figure 9A), suggesting circulating proteins can affect myocyte stiffness. Of the proteins increased in the DCM secretome, circulating sFRP1 has been shown to affect cellular stiffness (19, 20) and is a likely candidate for promoting cardiomyocyte stiffness. SOMAscan-based proteomics analysis identified a significant 4-fold increase in circulating sFRP1 (Figure 9B). Interestingly, sFRP1 transcripts are also increased in the pediatric DCM heart (based on RNA-Seq data; ref. 4); Figure 9C). To test if circulating sFRP1 promoted stiffness in NRVMs, AFM was performed on NRVMs treated with or without human sFRP1 (hsFRP1). As shown in Figure 9E, hsFRP1, but not MDK (Figure 9D), treatment of NRVMs resulted in a significant increase in stiffness when compared with untreated controls. hsFRP1 did not affect changes in the FGP (data not shown).

## Discussion

Focused studies and the development of animal models that recapitulate characteristics of adult DCM have resulted in a better understanding of the disease process and development of therapies specific for this population. In children, however, the assumption that the causes of DCM are similar to adults has contributed to limited improvement in outcomes in the pediatric population (6, 21). We and others have shown age-specific differences in the cardiac gene expression profile, presence of fibrosis and hypertrophy, and miRNA expression (4–6, 11, 12, 22). These differences highlight the importance of age-focused studies. However, a limitation to further advance our understanding of the myocellular mechanisms altered in pediatric DCM is the lack of in vivo or in vitro models of the disease. To circumvent this problem, our group developed an in vitro–based system where NRVMs are treated with DCM patient sera, which reproduces molecular characteristics of the pediatric failing DCM heart (11, 23). In this study, we further characterized global changes in gene expression in response to serum treatment and the circulating factors that contribute to pathological cardiomyocyte remodeling. Our multiomics analysis suggests ECM remodeling, specifically focal adhesion, is an important pathway affected by factors present in the pediatric DCM serum. In addition, we investigate specific factors present in the serum and show that circulating miRNAs were protective and did not promote pathologic remodeling, but that the circulating proteins had a substantial role in promoting detrimental effects of serum-circulating factors. Specifically, we show that MDK contributed to DCM serum–mediated activation of the FGP, and that serum depletion of MDK blunted serum-mediated upregulation of ANF and BNP. Furthermore, we show that DCM serum treatment of NRVMs induced cellular stiffness, and that sFRP1 protein levels were increased in serum and promotes stiffness in NRVMs.

### Protective RNAs were present in NF sera, whereas pathological proteins were present in DCM sera.

We have previously shown that EVs/exosomes or the resulting serum suspension can promote activation of the FGP in patients with DCM, and that sera from patients with DCM has fewer EVs/exosomes than sera from NF patients (11). Exosomes are naturally engineered for the selective loading of miRNAs and can act as paracrine carriers of circulating miRNAs (24). Interestingly, sera from our DCM population demonstrate an overwhelming downregulation of miRNAs when compared with age-matched NF controls (Figure 3A). Importantly, DNase treatment of NF serum did not show an appreciable change in FGP induction (Figure 2A). However, RNase treatment of NF serum resulted in a significant increase in the expression of BNP and ANF and a trend to downregulation of the αMyHC/βMyHC ratio (Figure 2B). The downregulation of DCM serum miRNAs suggest that circulating miRNAs were protective and that their downregulation contributed to pathologic remodeling. These results are in alignment with prior studies that showed that miRNAs derived from cardiac progenitor cells are cardioprotective (reviewed in ref. 25).

These results suggest that secretome factors, other than miRNAs, may be responsible for pathological remodeling, and that lower levels of miRNAs may contribute to these detrimental effects. Correspondingly, no additional changes in FGP were observed in response to RNase treatment of DCM serum. Although increased levels of circulating miRNAs have been associated with DCM in adults (26), the observed downregulation of circulating miRNAs suggest a unique age-dependent characteristic of the pediatric DCM population. To determine if proteins present in sera from patients with DCM could contribute to pathological remodeling, we treated NRVMs with patient sera with or without PTNK. Shown in Figure 4, PTNK treatment of DCM serum resulted in a reversal of the FGP. These results indicate that circulating proteins were important modulators of pathological gene expression and may have had a role in disease progression. In support of this, several proteins were upregulated in the serum of pediatric patients with DCM, and the role of these proteins in pathological remodeling needs to be further explored.

### Circulating MDK contributed to an increased expression of ANF and BNP.

Our results showed a dramatic increase in the levels of circulating MDK in the secretome of pediatric patients with DCM (Figure 6A). MDK is a heparin binding growth factor involved in development and disease. MDK activates several pathways, with STAT activation perhaps being the most recognized target of MDK (reviewed in ref. 27). In adults with HF, higher levels of circulating MDK are associated with worse cardiac function (28, 29); however, the role of MDK in cardiac pathology is not clear. As extensively discussed in a review we published recently, the effect of MDK in the heart is variable (27). Our results suggest that circulating MDK contributes to pathological changes in gene expression through increased expression of ANF and BNP. However, MDK did not affect expression of α-MyHC or β-MyHC, suggesting other circulating proteins are involved in regulating expression of these genes.

### ECM remodeling/focal adhesion pathways were upregulated in DCM serum–treated NRVMs, and in the human pediatric DCM circulating proteome and miRnome.

ECM remodeling has been shown to be altered in the pediatric failing heart (8). Transcriptome analysis of DCM serum–treated NRVMs showed an enrichment of genes involved in ECM remodeling and focal adhesion. Our analysis of circulating proteins also indicates an upregulation of pathways involved in ECM remodeling. Alterations in ECM remodeling affect cellular responses that are often mediated by integrin focal adhesion signaling, resulting in reorganization of actin filaments and cytoskeleton remodeling, which can result in cellular stiffness. Importantly, focal adhesion pathway is predicted to be dysregulated in serum-treated NRVMs, in the circulating proteome and in dysregulated circulating miRNAs. Others have shown that focal adhesion kinase phosphorylation can lead to increased ECM deposits and myocardial remodeling (30, 31). In Table 5, ECM remodeling, focal adhesion, and PI3k/AKT pathway are significantly upregulated in the circulating proteome of patients with DCM. Although PI3k/AKT signaling plays a large role in cardiomyocyte survival, proliferation, metabolism, and growth, it has also been shown to mediate ECM remodeling and cardiomyocyte stiffness via titin isoform switching (32, 33).

Importantly, we found an upregulation of focal adhesion and PI3k/AKT signaling pathways in the NRVM transcriptome, circulating miRNAs, and the circulating proteome. It is possible that the downregulation of circulating miRNAs contributes to an increase in the expression of genes associated with cardiomyocyte stiffness. In other words, circulating miRNAs may be protective by targeting expression of genes that contribute to cellular stiffness. In fact, miRNAs have been shown to regulate key components of myocardial remodeling including hypertrophy, fibrosis, and increased myocardial stiffness (34). Inhibition of miRNA-29, for example, increased collagen mRNA levels in vitro, and miRNA-29 negatively regulates mRNAs encoding collagens, fibrillins, elastins, and other ECM proteins (35). miRNA-29 was downregulated in pediatric DCM serum (Supplemental Table 3), similar to what we previously showed in pediatric DCM LV tissue (5). In summary, these results indicate that factors that can cause myocyte cytoskeleton remodeling are dysregulated in serum-treated NRVMs, in the patient proteome, and as a result of decreased miRNA levels, which may contribute to an increase in cellular stiffness. Importantly, predicted pathways altered in pediatric DCM hearts included regulation of actin cytoskeleton (4), supporting our finding that circulating factors affecting cytoskeleton remodeling can play an important role in pediatric DCM. Therefore, ECM remodeling and intracellular cytoskeletal components may be important in understanding pediatric DCM pathology. In support of these findings, an evaluation of pathways commonly altered in the serum-treated NRVM transcriptome and pediatric DCM transcriptome showed that the top overlapping pathways include pathways related to ECM remodeling, signaling by RHO GTPases. and collagen formation (Table 8).

### Exogenously delivered sFRP1 increased cellular stiffness in NRVMs.

Our results suggest that cardiomyocyte stiffness may be a common end result of changes in the pediatric DCM secretome. To test this hypothesis, we investigated if serum treatment of NRVMs could recapitulate cardiomyocyte stiffness by performing AFM measurements. Figure 9A shows increased cellular stiffness in NRVMs treated with DCM patient serum.

We sought to use our proteome data to further evaluate which upregulated proteins could be involved in promoting cellular stiffness. Of the proteins upregulated in the circulation, sFRP1 has been previously shown to promote cellular stiffness in human trabecular meshwork cells (HTMCs) isolated from donor corneoscleral rims (19). Importantly, sFRP1 is involved in promoting stress fiber and focal adhesion formation in trabecular meshwork (36). Additionally, based on our transcriptome data (details published in ref. 4), sFRP1 mRNA levels were significantly increased in DCM LV tissue (Figure 9C; ref. 4). sFRP1 is a part of the sFRP family, which are known Wnt antagonists (37). The Wnt/frizzled pathway is involved in cell-cell adhesion of cardiomyocytes, and endogenous sFRP1 has been shown to be necessary for normal myocardial development as overexpression can increase the development of heart muscle (37). Sklepkiewicz et al. showed that a sFRP1-KO mouse model displays ventricular dilation and hypertrophy along with cardiac fibrosis in aging animals. Notably these mice are normal at birth and develop cardiac abnormalities in older age (38). Additionally, sFRP1 was shown to attenuate cardiac dysfunction in transverse aortic constriction mice, where an AAV9 sFRP1 vector improves function by decreasing myocardial apoptosis (39). sFRPs have been shown to alleviate collagen deposition and fibrosis in cardiac tissue; however, systemic sFRP1 is significantly higher in patients with cardiovascular disease (40), and its increased circulating levels are associated with the development of cardiovascular diseases (41). Furthermore, treatment of cardiac progenitor cells with sFRP1 promote cellular senescence (42), and extracellular sFRP1 is associated with doxorubicin-induced cardiomyopathy (43). These results suggest that extracellular sFRP1 can have detrimental effects on cardiomyocytes. Given that sFRP1 increases stiffness in HTMC, we tested if sFRP1 could elicit the same response in NRVMs. Figure 9E shows that exogenously delivered sFRP1 increased cellular stiffness in NRVMs, consistent with findings in HTMC. These results suggest that extracellular sFRP1 may have had pathologic effects, and further studies are warranted.

### Conclusions.

A multiomic analysis of pediatric DCM secretome identified alterations in focal adhesion/regulation of actin cytoskeleton/ECM remodeling as a unifying theme in the transcriptome from serum-treated NRVMs, in circulating proteins, and in response to decreased levels of circulating miRNAs. We show that miRNAs present in NF serum were protective and prevented the upregulation of pathologic markers, and that differentially regulated circulating proteins had likely contributed to pathological changes in gene expression in pediatric patients with DCM. Lastly, we show that MDK contributed to serum-mediated changes in gene expression and sFRP1 contributed to cardiomyocyte stiffness, which warrants future studies to further explore their potential role in pathological remodeling and HF progression in the pediatric DCM population.

### Limitations.

There are limitations to this study (1). This is an in vitro study, and it is not clear if these factors would have similar effects in vivo. However, a previous study elegantly showed an effect of exosomes in the heart in vivo (15), which supports the role of circulating factors in pathologic remodeling (2). Due to the relative rarity of pediatric DCM, we are unable to determine the influence of age (infants vs. teenagers), the effect of HF duration, or the role of specific medications (3). We recognize that mRNA levels may not be representative of protein content or enzymatic activity (4). Although we evaluated the relative contribution of DNA, RNA, and proteins to pathologic responses and implicated MDK and sFRP1 in changes in gene expression and cardiomyocyte stiffness, several other proteins are upregulated in the secretome and may play a role in pathological changes in cardiomyocytes. Further studies are necessary to investigate the contribution of these proteins to cardiac pathology. Importantly, we did not investigate if these proteins were localized to EVs or are free-circulating factors (5). We did not find an association between MDK or sFRP1 levels and parameters of cardiovascular dysfunction (data not shown), which suggests that these circulating proteins may not be a good biomarker of disease progression/severity in children or that studies with a larger sample size are necessary to investigate their role as potential biomarkers in this population (6). This is a cross-species studies and we cannot discard the possibility that human serum may have effects on rat cells unrelated to the disease process. Serum from NF controls were added to all experiment to mitigate this potential issue. Last, the specific mechanisms involved in the response to circulating proteins have not been evaluated.

## Methods

### Human samples.

NF blood samples were from children (younger than 18 years of age) who had normal cardiac structure and function and/or no history of cardiac disease and were not brain dead. Patient blood samples (younger than 18 years of age) were included based on a clinical diagnosis of DCM as documented in the electronic medical record. The diagnosis of DCM was further supported by included DCM subjects having an ejection fraction less than or equal to 45% and/or left ventricular end diastolic dimension z score greater than or equal to 2. Patients on mechanical circulatory support were included only if support duration was not longer than approximately 1 month, because this duration of support did not affect the response to serum (data not shown). Blood samples were collected, processed, and stored at –80°C until use. Samples were aliquoted to avoid multiple freeze/thaws. Subjects were selected based on meeting inclusion criteria and with an effort to match the NF and DCM groups based on age and sex. Samples were not pooled for any of the experiments, except RNA-Seq, where RNA from 2 different plates were pooled. Blood was drawn prior to bypass to avoid possible heparin interference with the polymerase. Patients treated with heparin were on doses appropriate for thrombus prevention (0.2–0.7 U/mL), which are below the levels shown to inhibit the polymerase (44). Patient demographics are listed in Supplemental Table 1. Clinical characteristics and medications reflect time of sampling. Outcomes for each patient are listed in Supplemental Table 1.

### Cell culture: serum and recombinant MDK and sFRP1 treatment.

NRVMs were isolated from the ventricles of 1- to 2-day-old Sprague-Dawley rats (Charles River) by enzymatic digestions as previously described (45). Cells were plated at 160,000 cells/mL density. NRVMs were treated with 2% NF or DCM human serum for 48–72 hours as previously described (11), 100 ng/mL of human MDK (46, 47), or 1 μg/mL hsFRP1 (48, 49) recombinant protein (R&D Systems) for 72 hours.

### DNase, RNase, and PTNK serum treatment.

Serum sample aliquots (NF and DCM) were thawed on ice. Nuclease-free water (5 μL) was combined with serum (20 μL) to avoid volume loss due to heat/freeze protocol (methodology previously described in detail in ref. 16). We have previously shown (16) that this methodology substantially increases miRNA detection. Briefly, samples were heated at 65°C for 5 minutes and then flash-frozen in an ethanol/dry ice mixture. Heat/freeze cycle was repeated 2 more times. Samples were then treated with RNase or DNase. For DNase, 0.1 volume 10X DNase buffer plus 1.0 μL of DNase Turbo (2 U; Thermo Fisher Scientific) was combined with serum and incubated at 37°C for 1 hour. DNase was inactivated with 0.1 volume of DNase Inactivation Reagent (Thermo Fisher Scientific) and incubated at room temperature for 5 minutes. Samples were centrifuged at room temperature for 1.5 minutes at 10,000*g*. Supernatant was transferred to a new Eppendorf tube. For RNase, 1.5 μL of RNase (10 μg/mL; Thermo Fisher Scientific) was added to serum samples and incubated at 37°C for 30 minutes, followed by RNase inactivation at 65°C for 5 minutes. For PTNK, Proteinase K (Thermo Fisher Scientific) was added to serum to a final concentration of 100 μg/mL and incubated at 50°C for 1 hour. PTNK in the serum was inactivated with 100 μM of PMSF (Thermo Fisher Scientific).

### Transfection.

NRVMs were transfected with DNase- or RNase-treated serum. In a 12-well plate, 3 μL of Lipofectamine RNAiMAX (Thermo Fisher Scientific) was diluted in 50 μL of Opti-MEM (Thermo Fisher Scientific) reduced serum media, mixed, and incubated for 5 minutes. In parallel, 20 μL serum with or without DNase or RNase was added to 33 μL of Opti-MEM, mixed, and incubated for 5 minutes. The serum/Opti-MEM was combined with the Lipofectamine RNAiMAX/Opti-MEM and incubated for 5 minutes at room temperature. The serum–Lipofectamine RNAiMAX complex was then added to each appropriate well. Cells were harvested 72 hours after transfection.

### RNA extraction and real-time quantitative PCR.

NRVMs were homogenized in Qiazol (QIAGEN), and RNA was extracted and precipitated using the chloroform/isopropanol method optimized by our laboratory (50). cDNA was synthesized by using the Applied Biosystems High Capacity cDNA Synthesis Kit (Thermo Fisher Scientific) according to the manufacturer’s instructions. Gene expression was measured by real-time quantitative PCR (RT-qPCR) as previously described (51), with Power Sybr Green PCR Master Mix (Life Technologies). Expression levels of all transcripts were normalized to 18S rRNA, and no difference in the expression of 18S between groups was appreciated (data not shown). All data were log2 transformed. RT-qPCR primer sequences are listed in Garcia et al. (23).

### RNA-Seq analysis.

RNA was extracted from serum-treated NRVMs as described above, quantified using Qubit (Biotium) fluorometric quantitation, and assessed for quality using the Agilent Bioanalyzer Nano RNA Chip (Agilent). Samples with an RNA integrity number greater than 9 were considered to be high quality and suitable for RNA-Seq. 1X150 directional mRNA sequencing was performed using an Illumina hiSEQ4000 (HT Mode), resulting in an average of 38–44 million mapped reads per sample. The raw data were subjected to QC analyses using the FastQC tool (http://www.bioinformatics.babraham.ac.uk/projects/fastqc/). Reads were aligned to the reference genome *R*. *norvegicus* (Rnor_5.0) using gsnap (52). Fragments per kilobase of exon per million mapped reads values were calculated using Cufflinks (53) for each sample (*n* = 6 NF serum–treated NRVMs and *n* = 6 DCM serum–treated NRVMs, from 3 different NRVM preps; 2 independent RNA preparations from each prep were pooled). The transcripts were normalized to the NF group within each NRVM prep and transcripts with low variance (<0.1) across samples were removed. After removal of low variance genes, 4079 genes remained. Statistically significant changes in gene expression were calculated using log2 transformed data and a 2-tailed Welch’s *t* test assuming equal variance with a *P* value adjusted for false discovery rate, *q* < 0.10. Gene ID, fold change, and *q* value are presented in Supplemental Table 2. RNA-Seq data were downloaded from the GEO database using accession number GSE181051 (https://www.ncbi.nlm.nih.gov/geo/query/acc.cgi?acc=GSE181051).

### MegaPlex pool and TaqMan low-density miRNA array: miRnome analysis.

Samples (*n* = 12 NF and *n* = 32 DCM) were prepared by taking serum aliquots and performing 3 freeze/thaw cycles as previously described (16). A modified MegaPlex pool and TaqMan low-density arrays were performed on serum samples per the manufacturer’s protocol (Applied Biosystems) as previously described (16). miRNAs with Ct values greater than 35 or Amp score greater than 0.9 were excluded from the analysis. miRNA expression was normalized to miRNA-320, which is the least variable miRNA in over 400 pediatric samples (data not shown). miRNAs expressed in 75% of the samples were included in the analysis. miRNAs with a *q* value less than 0.10 (Wilcoxon’s test) were further evaluated and are shown in Supplemental Table 3. miRNA array data were downloaded from the GEO database using accession number GSE181051 (https://www.ncbi.nlm.nih.gov/geo/query/acc.cgi?acc=GSE181051).

### SOMAscan: proteome analysis.

Plasma from NF (*n* = 4) and DCM (*n* = 8) patients were prepared by collecting whole blood in vacutainers containing anticoagulant citrate dextrose. Samples were centrifuged at 2200*g* for 15 minutes at room temperature. The plasma layer was then aliquoted and stored at –80°C until use. SOMAscan Platform experiments were performed by the University of Colorado Genomics core as described by Candia et al. (54). Calibration normalizations resulted in 1141 detected proteins. Welch’s 2-tailed *t* test with an adjusted FDR of *q* < 0.10 was used as cutoff for significant dysregulated proteins. Protein ID, fold change, and *q* value are presented in Supplemental Table 4. SOMAscan data were downloaded from the GEO database using accession number GSE181051 (https://www.ncbi.nlm.nih.gov/geo/query/acc.cgi?acc=GSE181051).

### Differential expression representation of ontology data: transcriptome, proteome, and miRnome.

Volcano plot and hierarchical clustering/heatmap generation was performed using R (The R Foundation; ref. 55). IPA and Metascape platforms were used to investigate molecular pathways and toxicity functions associated with significant dysregulated genes. Predicted pathways affected by circulating miRNAs were investigated using miRPathDB (18).

### AFM assessment of cellular stiffness.

NRVMs were treated with DCM or NF serum, with recombinant hsFRP1, or recombinant human MDK, or water for 72 hours as described above. After 72 hours, cells were treated with 1 mg/mL of 2,3-Butanedione monoxime 1 hour before AFM measurements to stop cardiomyocyte beating. The methodology used for the present work was based on previous publications (48, 52), with some modifications. Cells were analyzed using a JPK NanoWizard 4a BioScience AFM with a PetriDishHeater tool set at 37°C. Force Spectroscopy mode was used to determine the nanomechanical properties of NRVMs. MLCT AFM probes were used, with a pyramidal Si3N4 35° curvature radius tip (Bruker). The cantilever spring constants were systematically measured (0.01–0.02 N/m) using the thermal tune method. The maximal force applied to the cell was limited to 1.5 nN, with 5 μm of Z piezo.

All physical and physiological cues regarding AFM analysis and sample preparation were kept constant across all samples. Cells were monitored and their morphological details observed, and an optical light microscope was used for cell selection. Only well-spread cells were investigated; those with a round shape and a dark edge were not included in the analysis. An average of 20 to 30 cells were analyzed per group from 3 independent serum-treated or hsFRP1-treated NRVM preps. The Hertz-Sneddon model (56–58) was used to obtain the elasticity of cells studied using the JPK data processing software.

### Statistics.

Statistical analyses for all multiomics data are described in each omics section. All other analyses were performed using GraphPad Prism 8 (GraphPad Software), and significance threshold was set a priori at *P* < 0.05. All RT-qPCR, SOMAscan, and tissue transcriptome MDK and sFRP1 data were log2 transformed and analyzed by unpaired 2-tailed *t* test. AFM data were checked for normality using Shapiro-Wilk. If not normally distributed, the Mann-Whitney *U* test was used. Quantitative results are presented as mean ± SEM. One-way ANOVA was used for comparisons of 3 or more normally distributed groups, and Sidak’s multiple comparison tests were performed when analyzing 3 or more interdependent groups. Each appropriate statistical test is reported in the figure legends.

### Study approval.

Blood was collected from subjects who gave informed consent to the IRB-approved Investigations of Pediatric Heart Disease blood bank at the University of Colorado, Denver. Human tissue collection was approved by the University of Colorado Anschutz Medical Campus COMIRB. This study complies with the Declaration of Helsinki. Written informed consent was obtained from each patient or family of the donors. Animal experiments were in compliance with the *Guide for the Care and Use of Laboratory Animals* (National Academies Press, 2011) and approved by the IACUC of the University of Colorado Anschutz Medical Campus. All animal protocols are in accordance with PHS Animal Welfare Assurance (A3269-01).

## Author contributions

DAJ performed experiments, data analysis, data interpretation, and manuscript writing. JPDS performed experiments, data analysis, data interpretation, and manuscript writing. AMG performed experiments, data analysis, data interpretation, and manuscript editing. XJ performed experiments, data analysis, and data interpretation. AKF performed multiomic analysis and manuscript editing. LST performed data analysis. TL performed experiments and data analysis. BP performed experiments, manuscript editing, and data analysis. CAM compiled the patient demographics/clinical characteristic table. KN performed sample preparation and data analysis. AK performed sample preparation and data analysis. OS supervised AFM experiments and data interpretation. MRGT supervised AFM experiments. SDM performed experimental planning, patient selection, and manuscript writing. BLS performed experimental planning and manuscript editing. CCS performed study conception, experimental planning, data analysis, and manuscript writing. All authors reviewed and approved the manuscript.

## Supplementary Material

Supplemental data

## Figures and Tables

**Table 1:**
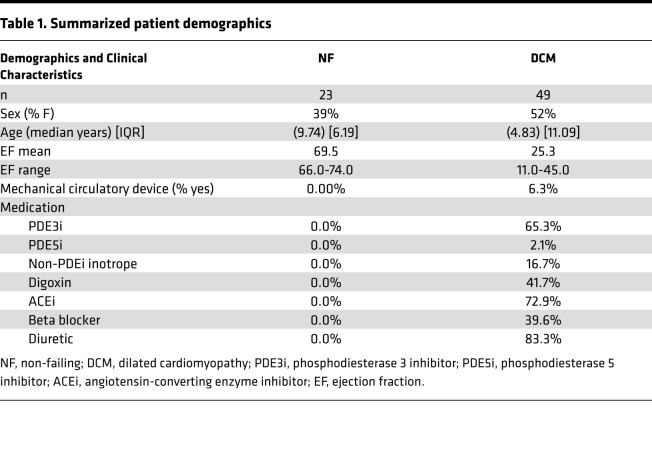
Summarized patient demographics

**Table 8:**
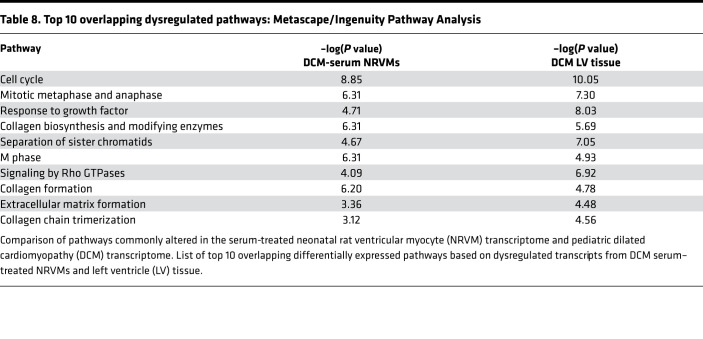
Top 10 overlapping dysregulated pathways: Metascape/Ingenuity Pathway Analysis

**Table 7:**
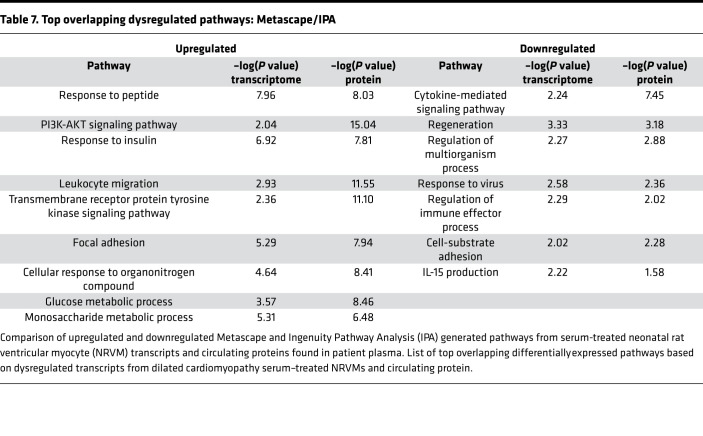
Top overlapping dysregulated pathways: Metascape/IPA

**Table 6:**
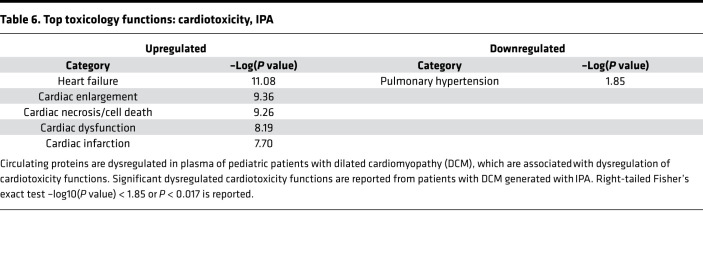
Top toxicology functions: cardiotoxicity, IPA

**Table 2:**
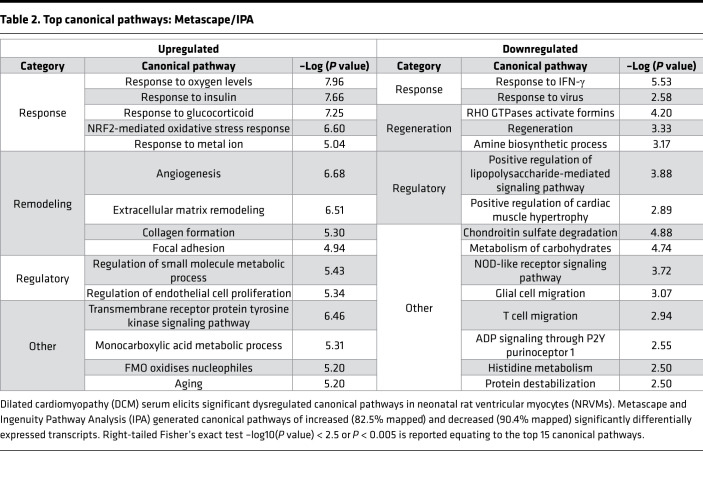
Top canonical pathways: Metascape/IPA

**Table 3:**
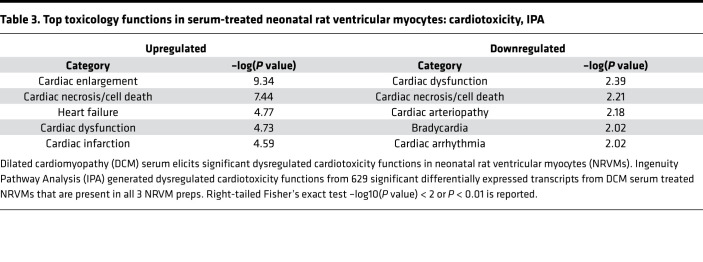
Top toxicology functions in serum-treated neonatal rat ventricular myocytes: cardiotoxicity, IPA

**Table 4:**
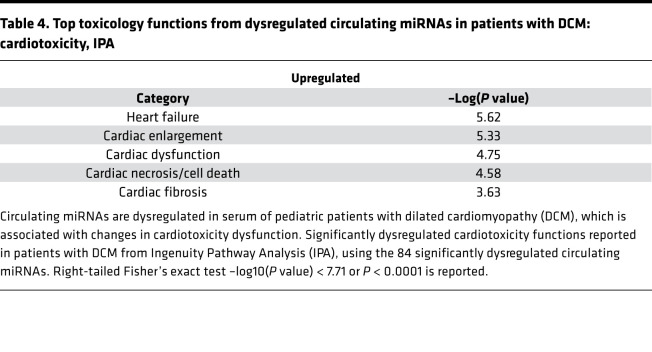
Top toxicology functions from dysregulated circulating miRNAs in patients with DCM: cardiotoxicity, IPA

**Table 5:**
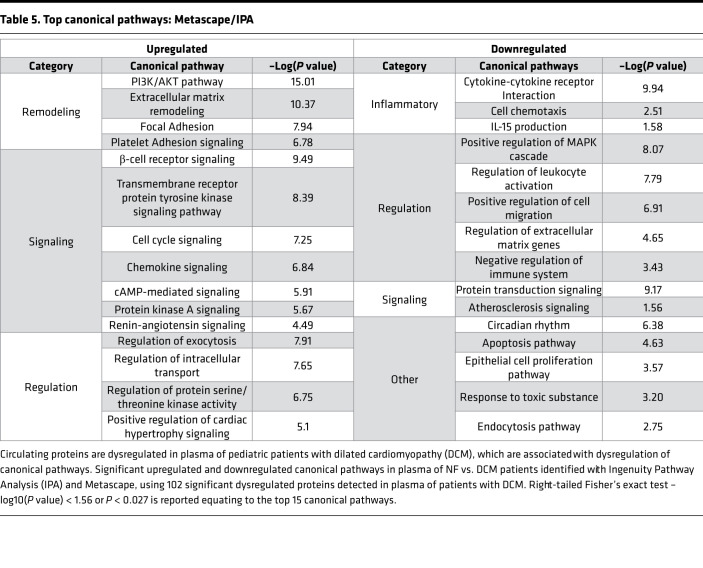
Top canonical pathways: Metascape/IPA

## Version 1 08/12/2021

In-Press Preview

## Version 2 10/08/2021

Electronic publication
