# Supplementary material for: Serum circulating proteins from pediatric patients with dilated cardiomyopathy cause pathologic remodeling and cardiomyocyte stiffness
Source: JCI Insight. 2021 Oct 8;6(19):e148637. doi: 10.1172/jci.insight.148637 (PMC8525651; doi:10.1172/jci.insight.148637)
Supplement: Supplemental data [file jciinsight-6-148637-s295.pdf]

ID	Diagnosis	Age (yrs)	Sex	PDE3i	PDE5i	Non-PDEi Inotrope*	Digoxin	ACEi	β-Blocker	Diuretic	EF	Etiology of DCM	Mechanical Circulatory Support	RNA-seq from NRVMs	RNase DNase RT-qPCR	PTK RT-qPCR	miRNA array	SOMAscan	AFM
**1**	NF	5.64	F	N	N	N	N	N	N	N	SN		N				**X**		**X**
**2**	NF	12.55	M	N	N	N	N	N	N	N	74		N		**X**				
**3**	NF	12.78	F	N	N	N	N	N	N	N	SN		N			**X**			
**4**	NF	5.45	F	N	N	N	N	N	N	N	41 (FS)		N				**X**		
**5**	NF	4.05	M	N	N	N	N	N	N	N	69		N				**X**		
**6**	NF	5.39	M	N	N	N	N	N	N	N	-		N		**X**	**X**	**X**		
**7**	NF	4.12	M	N	N	N	N	N	N	N	66		N		**X**	**X**	**X**		
**8**	NF	9.89	F	N	N	N	N	N	N	N	-		N	**X**					
**9**	NF	4.34	M	N	N	N	N	N	N	N	-		N	**X**					
**10**	NF	9.74	M	N	N	N	N	N	N	N	-		N	**X**					
**11**	NF	11.83	F	N	N	N	N	N	N	N	-		N	**X**					
**12**	NF	10.79	F	N	N	N	N	N	N	N	-		N			**X**			
**13**	NF	7.72	M	N	N	N	N	N	N	N	-		N					**X**	
**14**	NF	7.19	M	N	N	N	N	N	N	N	-		N					**X**	
**15**	NF	10.10	M	N	N	N	N	N	N	N	-		N					**X**	
**16**	NF	9.09	M	N	N	N	N	N	N	N	-		N					**X**	
**17**	NF	10.26	M	N	N	N	N	N	N	N	37(FS)		N				**X**		
**18**	NF	11.74	F	N	N	N	N	N	N	N	-		N				**X**		
**19**	NF	4.12	M	N	N	N	N	N	N	N	-		N				**X**		
**20**	NF	11.92	F	NA	NA	NA	NA	NA	NA	NA	-		N				**X**		
**21**	NF	0.88	M	N	N	N	N	N	N	N	-		N				**X**		
**22**	NF	13.56	F	N	N	N	N	N	N	N	-		N				**X**		
**23**	NF	11.47	M	N	N	N	N	N	N	N	-		N				**X**		
**NF**	**n = 23**	**8.46**	**39% F**	**0.0%**	**0.0%**	**0.0%**	**0.0%**	**0.0%**	**0.0%**	**0.0%**	**69.5**	**NA**	**0.0%**	**17.4%%**	**13.0%**	**17.4%**	**52.0%**	**17.4%**	**4.3%**
**24**	DCM	15.82	M	Y	N	Y	N	N	N	N	20	IDC	N				**X**		
**25**	DCM	18.92	F	N	N	N	N	Y	Y, nonselective	Y	21(FS)	Sarcomere variant	N			**X**			
**26**	DCM	6.79	M	N	N	N	Y	Y	Y, nonselective	Y	45	In utero ischemia	N		**X**				
**27**	DCM	17.34	M	Y	Y	Y	Y	Y	Y, nonselective	Y	5(FS)	IDC	Y		**X**				
**28**	DCM	3.59	F	Y	N	N	N	Y	N	Y	28	IDC	N	**X**			**X**		
**29**	DCM	13.48	M	Y	N	N	N	N	Y, nonselective	N	23(FS)	IDC	N	**X**					
**30**	DCM	9.01	M	Y	N	Y	N	Y	Y, β1 selective	Y	NA	IDC	Y	**X**	**X**				
**31**	DCM	5.20	M	Y	N	N	N	Y	Y, β1 selective	Y	16	IDC	N	**X**			**X**	**X**	
**32**	DCM	2.74	F	N	N	N	Y	Y	Y, nonselective	N	31	IDC	N	**X**					
**33**	DCM	2.46	F	Y	N	N	Y	Y	N	Y	41	IDC	N	**X**			**X**		
**34**	DCM	6.48	F	N	N	N	N	Y	N	Y	25	IDC	N		**X**				
**35**	DCM	16.57	M	N	N	N	Y	Y	Y, nonselective	Y	24	IDC	N		**X**				
**36**	DCM	17.35	M	N	N	N	N	Y	N	Y	17	IDC	N		**X**				**X**
**37**	DCM	0.16	M	N	N	N	N	Y	N	N	32	IDC	N		**X**				
**38**	DCM	0.57	M	Y	N	N	N	Y	N	Y	27	IDC	N		**X**		**X**		

**39**	DCM	5.93	M	N	N	N	Y	Y	Y, nonselective	Y	41	In utero ischemia	N		**X**	X			
**40**	DCM	15.75	M	Y	N	Y	Y	N	N	Y	15	IDC	Y			**X**			
**41**	DCM	18.44	M	Y	N	N	Y	Y	Y, β1 selective	N	37	IDC	N			**X**			
**42**	DCM	0.16	F	Y	N	N	N	N	N	Y	11	Sarcomere variant	N					**X**	
**43**	DCM	10.01	F	N	N	N	N	Y	Y, nonselective	Y	22	IDC	N					**X**	
**44**	DCM	17.16	M	Y	N	N	Y	Y	N	Y	24	IDC	N					**X**	
**45**	DCM	3.50	F	N	N	N	N	Y	N	Y	29	IDC	N					**X**	
**46**	DCM	0.45	F	Y	N	N	N	N	Y, β1 selective	Y	20	IDC	N				**X**	**X**	
**47**	DCM	11.61	M	Y	N	N	N	Y	N	Y	20	IDC	N				**X**	**X**	
**48**	DCM	0.73	F	Y	N	N	Y	N	N	Y	20	IDC	N				**X**	**X**	
**49**	DCM	2.06	F	Y	N	N	N	N	N	N	17	Familial	N				**X**		
**50**	DCM	3.88	M	N	N	N	N	Y	N	N	43	IDC	N				**X**		
**51**	DCM	0.09	F	Y	N	Y	Y	N	N	Y	15	IDC	N				**X**		
**52**	DCM	14.60	F	Y	N	Y	N	N	N	Y	4(FS)	IDC	N				**X**		
**53**	DCM	18.40	F	Y	N	N	Y	Y	N	Y	37	IDC	N				**X**		
**54**	DCM	4.83	M	N	N	N	N	Y	N	N	21	IDC	N				**X**		
**55**	DCM	0.70	F	Y	N	N	N	N	N	Y	16	IDC	N				**X**		
**56**	DCM	0.20	F	N	N	N	N	Y	N	Y	14	IDC	N				**X**		
**57**	DCM	13.29	F	Y	N	N	N	N	N	Y	27	IDC	N				**X**		
**58**	DCM	3.15	M	N	N	N	Y	Y	Y, nonselective	Y	32	IDC	N				**X**		
**59**	DCM	0.22	M	Y	N	N	Y	Y	N	Y	-	IDC	N				**X**		
**60**	DCM	0.26	M	Y	N	N	Y	Y	N	Y	-	IDC	N				**X**		
**61**	DCM	0.39	F	Y	N	Y	N	N	N	Y	17	IDC	N				**X**		
**62**	DCM	2.27	F	N	N	N	Y	Y	N	Y	30	IDC	N				**X**		
**63**	DCM	2.83	F	Y	N	N	Y	Y	N	Y	37	Familial	N				**X**		
**64**	DCM	3.40	F	Y	N	N	N	N	N	N	30	IDC	N				**X**		
**65**	DCM	10.57	F	N	N	N	Y	Y	Y, nonselective	Y	21(FS)	IDC	N				**X**		
**66**	DCM	10.63	F	Y	N	N	Y	Y	Y, nonselective	Y	22(FS)	IDC	N				**X**		
**67**	DCM	2.86	M	Y	N	N	N	Y	N	Y	14	IDC	N				**X**		
**68**	DCM	9.76	M	Y	N	N	N	Y	N	Y	21	IDC	N				**X**		
**69**	DCM	4.14	F	Y	N	N	Y	Y	Y, nonselective	Y	32	IDC	N				**X**		
**70**	DCM	2.19	M	N	N	N	N	Y	Y, nonselective	Y	24	IDC	N				**X**		
**71**	DCM	6.68	F	Y	N	Y	N	N	N	Y	16	IDC	N				**X**		
**72**	DCM	17.61	M	Y	N	N	N	Y	N	Y	25	Anthracycline-induced DCM	N				**X**		
**DCM**	**n = 48**	**7.25**	**52% F**	**65.3%**	**2.1%**	**16.7%**	**41.7%**	**72.9%**	**39.6%**	**83.3%**	**25.3**		**6.3%**	**12.5%**	**18.8%**	**8.3%**	**66.7%**	**16.7%**	**4.2%**

Table S1A: Pediatric Patient Characteristics. ID = identification, NF = non-failing, DCM= dilated cardiomyopathy, Average Age, M = male, F = female, PDE3i = phosphodiesterase 3 inhibitor, PDE5i = phosphodiesterase 5 inhibitor, ACEi = Angiotensin-Converting Enzyme Inhibitor, EF = ejection fraction, FS = fractional shortening, IDC = idiopathic dilated cardiomyopathy, SN = subjectively normal. *Inotropes include: dobutamine, dopamine, epinephrine, norepinephrine.

ID	Diagnosis	Outcome	MRI	Fibrosis on MRI	LVEDd	LVEDd z-score	LVEDVi	E/e'	E/e' z-score	TR	TAPSE	LA Volume indexed	Other medications
1	NF												
2	NF												
3	NF												
4	NF												
5	NF												
6	NF												
7	NF												
8	NF												
9	NF												
10	NF												
11	NF												
12	NF												
13	NF												
14	NF												
15	NF												
16	NF												
17	NF												
18	NF												
19	NF												
20	NF												
21	NF												
22	NF												
23	NF												
**NF**	**n = 23**	NA	NA	NA	NA	NA	NA	NA	NA	NA	NA	NA	NA
24	DCM	TX	Y	N	7.6	8.2	183.0	16.2	7.3	NA	2.2	NA	Calcium chloride, enoxaprin
25	DCM	Stable DCM	N	NA	5.8	1.1	56.0	4.5	-0.3	NA	1.6	17.4	N
26	DCM	TX	N	NA	5.4	8.2	129.0	11.9	4.2	NA	2.9	224.5	Aspirin, aldactone
27	DCM	TX	N	NA	7.7	7.2	73.0	NA	NA	3.0	NA	86.0	Warfarin, ASA
28	DCM	TX	N	NA	5.7	10.4	186.0	NA	NA	3.1	NA	NA	Selenium, folic acid
29	DCM	Died	N	NA	7.4	NA	99.9	NA	NA	3.2	NA	NA	Amiodarone, heparin
30	DCM	Tx	N	NA	4.4	2.2	58.2	7.6	NA	2.3	0.8	NA	Warfarin, zosyn, Enoxaparin, vancomycin, dipyridamole, megestrol acetate, clonazepam, lansoprazole, zyrtec
31	DCM	TX	N	NA	8.2	30.0	NA	9.9	NA	NA	1.3	91.0	Coumadin, warfarin
32	DCM	TX	N	NA	4.5	NA	67.9	NA	NA	NA	NA	NA	Carnitine
33	DCM	TX	N	NA	4.3	NA	135.8	NA	NA	4.0	NA	NA	Synthroid
34	DCM	TX	N	NA	6.5	11.9	173.8	11.5	NA	NA	9.0	40.7	N
35	DCM	TX	N	NA	6.8	5.3	153.0	12.1	NA	4.3	1.6	62.6	Carnitine-B5-B6, coenzyme Q10
36	DCM	Stable DCM	Y	Y	6.6	3.2	92.0	7.7	1.7	3.1	0.9	21.3	N
37	DCM	Stable DCM	N	NA	2.9	2.9	78.0	10.2	1.4	NA	1.2	15.8	N
38	DCM	Tx	N	NA	6.0	25.6	NA	15.0	NA	NA	1.3	29.5	N
39	DCM	Tx	N	NA	5.3	8.4	149.0	11.9	4.1	NA	1.9	187.8	N
40	DCM	Tx	N	NA	5.9	4.8	102.0	13.2	5.4	2.6	1.0	40.5	Heparin
41	DCM	Tx	N	NA	8.3	10.1	202.0	22.6	11.6	3.3	2.7	143.4	Nitroprusside sodium
42	DCM	Tx	N	NA	3.8	9.0	173.8	NA	NA	2.3	NA	28.5	Precedex

**43**	DCM	Stable DCM	N	NA	7.4	11.8	266.9	8.2	NA	3.6	0.7	173.6	Flovent, metolazone
**44**	DCM	Tx	Y	Y	8.6	9.6	439.7	11.6	NA	3.3	1.3	55.8	Calcium carbonate
**45**	DCM	Tx	N	NA	5.7	10.3	152.4	NA	NA	NA	2.1	70.2	N
**46**	DCM	TX	N	NA	3.8	7.1	120.0	NA	NA	NA	0.8	13.3	Amiodarone, heparin, zantac, metoprolol
**47**	DCM	TX	Y	N	7.3	10.0	156.0	9.8	NA	3.3	1.2	34.9	Coumadin, zoloft, periactin, prevacid
**48**	DCM	TX	N	NA	4.7	10.6	170.0	17.8	NA	2.9	1.8	30.6	Lansoprazole
**49**	DCM	Tx	N	NA	NA	NA	NA	NA	NA	NA	NA	NA	
**50**	DCM	Tx	N	NA	4.0	-0.6	NA	NA	NA	4.3	NA	NA	
**51**	DCM	Died	N	NA	2.0	NA	NA	NA	NA	2.8	NA	NA	Heparin
**52**	DCM	Tx	N	NA	59.4	NA	NA	NA	NA	2.1	NA	NA	Heparin
**53**	DCM	TX	N	NA	7.4	7.0	182.0	14.5	6.3	3.0	1.5	129.5	N
**54**	DCM	Tx	N	NA	7.2	14.6	NA	18.3	NA	NA	1.6	50.6	Enoxparin, warfarin
**55**	DCM	TX	N	NA	5.0	12.0	56.3	15.3	NA	3.7	0.6	35.0	N
**56**	DCM	Tx	N	NA	3.3	6.2	28.0	NA	NA	3.4	1.1	31.2	N
**57**	DCM	Tx	N	NA	6.8	6.5	124.1	5.0	NA	3.2	1.6	79.3	N
**58**	DCM	Stable DCM	N	NA	4.6	6.3	92.0	13.2	4.8	NA	0.9	25.2	Carnitine
**59**	DCM	Tx	N	NA	NA	NA	NA	NA	NA	NA	NA	NA	N
**60**	DCM	TX	N	NA	NA	NA	NA	NA	NA	NA	NA	NA	N
**61**	DCM	TX	N	NA	NA	NA	NA	NA	NA	NA	NA	NA	N
**62**	DCM	TX	N	NA	4.7	NA	NA	NA	NA	4.2	NA	NA	Enoxaparin, synthroid
**63**	DCM	TX	N	NA	NA	NA	NA	NA	NA	NA	NA	NA	N
**64**	DCM	TX	N	NA	5.5	9.3	157.6	8.6	NA	3.4	NA	NA	Epogen, heparin
**65**	DCM	TX	N	NA	6.3	NA	NA	NA	NA	3.5	NA	NA	Amiodarone, coumadin
**66**	DCM	TX	N	NA	NA	NA	NA	NA	NA	NA	NA	NA	Amiodarone, coumadin
**67**	DCM	Tx	N	NA	4.5	5.3	NA	NA	NA	NA	NA	NA	Amiodarone, heparin, colace
**68**	DCM	TX	N	NA	5.9	6.7	112.2	16.0	NA	3.1	NA	NA	Amiodarone
**69**	DCM	TX	N	NA	3.1	5.2	63.9	NA	NA	3.8	NA	NA	Carnitine, coumadin
**70**	DCM	Tx	N	NA	4.6	6.2	178.0	NA	NA	NA	1.0	47.4	N
**71**	DCM	Tx	N	NA	6.9	14.0	224.9	NA	NA	2.8	1.3	109.9	heparin
**72**	DCM	Died	N	NA	6.7	3.0	53.4	NA	NA	2.7	NA	NA	N
**DCM**	**n = 48**		**8.30%**	**4.10%**	**6.9**	**8.6**	**137.9**	**12.2**	**4.7**	**3.2**	**1.7**	**69.5**	

Table S1B: Pediatric Patient Characteristics. ID = identification, NF = non-failing, DCM= dilated cardiomyopathy, Tx = transplant, LVEDd = Left ventricle end diastolic dimension, LVEDVi = Left ventricle end diastolic volume index, TR = tricuspid regurgitation, TAPSE = tricuspid annular plane systolic excursion, LA = left atria

Gene	foldChange	qvalue
1700092M07Rik	-0.5820193	0.03607622
AABR06008596.1	0.30506369	0.08076176
AABR06008986.1	-0.6653292	0.00028621
AABR06009537.3	0.56970007	0.07810388
AABR06009704.1	0.48727657	0.06459121
AABR06010645.1	0.88604787	0.03099922
AABR06011657.1	0.61725402	0.04612819
AABR06011847.1	0.6120324	0.05593551
AABR06013548.1	0.64991911	0.08895145
AABR06018852.1	0.64149928	0.06647063
AABR06024257.1	0.98001471	0.09743331
AABR06024265.1	0.42654716	0.0286959
AABR06027333.1	-0.4807441	0.06993342
AABR06028812.1	0.21682207	0.09288793
AABR06030862.1	-0.7300784	0.05631075
AABR06031707.1	-0.2173999	0.03890191
AABR06032013.1	0.45746651	0.01521807
AABR06032497.1	0.55106798	0.06645036
AABR06033179.1	0.47608213	0.08597666
AABR06034498.1	0.237606	0.04258825
AABR06036827.1	0.47889986	0.0262608
AABR06037200.1	0.41701365	0.07187697
AABR06037396.2	0.71825908	0.02563043
AABR06037778.1	2.4428999	0.0301873
AABR06039905.2	0.49876088	0.01521807
AABR06041255.1	-0.2312268	0.07113158
AABR06041388.1	0.84585489	0.02029366
AABR06043079.1	0.26671899	0.036405
AABR06054504.1	0.34871147	0.0262608
AABR06060231.1	1.00965187	0.03536404
AABR06060553.1	0.59607422	0.08670538
AABR06064824.1	0.55647815	0.03271195
AABR06065772.1	0.2952592	1.40E-07
AABR06066473.1	0.53708284	0.05835947
AABR06067081.1	0.86270308	0.0186233
AABR06079164.1	-0.3194946	0.05210007
AABR06080116.1	0.27959679	0.04017695
AABR06087049.1	0.57190374	0.06459121
AABR06087448.1	-0.48931	0.03607622
AABR06087544.1	0.76292838	0.03984483
AABR06088345.1	-0.6912209	0.02299134
AABR06089937.1	0.40011321	0.05760186
AABR06091761.1	0.34917635	0.07055603
AABR06099253.1	0.43869865	0.09255713
AABR06101044.1	0.70664304	0.06369604
Abhd14a	0.47201439	0.03236703
Abra	0.72675436	0.06560473
Ace3	1.00836757	0.09178405
Acer2	0.80834036	0.02059932
Acsbg1	2.46330077	0.02266576
Adamts12	0.85910737	0.08853583
Adamts8	0.06891287	0.06459121
Adamtsl3	-0.9469483	0.09032371
Adm	1.06699832	0.05664271
Agap1	0.48783693	0.05276295
Agl	-0.0902228	0.0591079
Ago2	0.24628938	0.036405
Aldh16a1	-0.3864781	0.00100253
Aldh1a3	-0.687128	0.09178405
Amz1	0.50243562	0.0146344

Ap1s2	-0.6842422	0.01521807
Ap1s3	-0.8633124	0.03984483
Apln	0.60708746	0.07894439
APLP1	-0.2754078	0.02699214
Areg	-0.4315267	0.08751081
Arhgap44	0.34960631	0.00374774
Arhgef3	-0.2406227	0.03796992
Arid5b	-0.4400196	0.06221291
Arl10	-0.4091668	0.07113158
Arl16	0.42593332	0.05778509
Arrdc2	0.77831759	0.03706967
Arsb	-0.7152664	0.09294903
Asph	-0.3818946	0.06463954
Atf6	0.46544839	0.05965958
Atp1b2	-0.9452633	0.09196767
Atp6v1g2	0.35262426	0.07227466
Avpi1	0.55478739	0.0843179
Axl	0.30574834	0.06930225
Azin1	-0.5551692	0.06993342
Azin2	-0.2467124	0.07142511
B4galt1	0.27113307	0.06459121
Bach1	0.43137358	0.05276295
Batf3	-0.3842638	0.07123019
Bcl2l1	0.58748371	0.08903586
Bik	-0.473827	0.09228086
Birc2	0.38556693	0.09294903
Birc3	0.16153786	0.08398838
Bmp2	0.22582957	0.02802798
Bmp6	0.49205567	0.05840574
C1qtnf1	0.80572326	0.01617524
C1r	0.68615094	0.01521807
C1s	0.74086347	0.08014955
C3	0.54728109	0.06107367
C5ar2	0.63631748	0.0973167
Camk2d	-0.5393343	0.04991324
Capn10	0.96265328	0.0437405
Car12	0.80262844	0.04112849
Carns1	-0.4279483	0.09377108
Casp7	-0.4063762	0.03960295
Casq1	0.17429747	0.04294003
Ccdc167	-0.6253859	0.09228086
Ccdc69	-0.2386744	0.00293291
Ccdc88c	-0.7702825	0.0841198
Ccl19	0.2257386	0.09768936
Ccl2	-0.8524976	0.0973167
Ccni	-0.2578108	0.07291425
Cd180	-0.6662861	0.06993342
Cd200r1	-0.2743858	0.05197438
Cd82	-0.2425165	0.0973167
Cdc25b	1.12447091	0.09308813
Cdca3	-0.6806821	0.02244641
Cdk2ap1	-0.3159433	0.07002721
Cdk6	0.69188064	0.01519568
Cdk9	-0.1540321	0.0263101
Cemip	-1.2262629	0.06993342
Cenph	-0.8007793	0.04346164
Cenpl	-0.4817717	0.00410302
Cenpm	-0.4266266	0.09321958
Cgref1	0.3159183	0.02028264
Chchd4	0.2826683	0.06713608
Chka	0.61986676	0.02891881

Chsy1	-0.5718225	0.07227466
Cited2	0.93650935	0.06369604
Ckap4	0.15026359	0.04823426
Ckmt2	1.99586656	0.00923005
Clcf1	-0.2715824	0.01426068
Cldn1	2.08032343	0.00691365
Cldn11	0.29981895	0.03984483
Cldn3	-0.3585221	0.09819598
Clec14a	0.14201565	0.01807383
Clic5	0.72459232	0.06463954
Clip4	-0.7893279	0.05824917
Clstn2	1.13215099	0.07278171
Cmklr1	1.11768333	0.04346164
Col4a1	0.52736354	0.02581162
Col4a2	0.49075444	0.0064057
Col6a1	0.89038304	0.04842385
Col6a2	0.90041934	0.00738241
Col7a1	0.76729547	0.03706967
Colq	0.434976	0.09026944
Corin	0.82232083	0.09308813
Cp	0.58502431	0.09606628
Cped1	0.52807385	0.03163937
Cpq	-0.3815775	0.01763871
Cpt1b	0.5428257	0.01753474
Cptp	0.38640775	0.00621992
Cpxm1	-0.5972174	0.06300925
Cpz	0.52788958	0.04084585
Creld2	-0.1992273	0.0146344
Crispld2	1.83552563	0.04476055
Crlf1	2.05286212	0.00964473
Cryab	0.65859875	0.09819598
Crygd	-1.1336233	0.04279455
Crym	-0.2482506	0.07101723
Csdc2	1.00128964	0.09951955
Cst3	0.57063524	0.0146344
Cstb	0.1542813	0.00139495
Cstf3	0.65277325	0.07520931
Ctrc	0.60125826	0.09945171
Cxcl11	-0.9390228	0.09672219
Cxcl6	-0.6358025	0.01063713
Cxcr4	0.66490222	0.00616785
Cyp26b1	0.75484059	0.0301873
Cyp2d4	0.58268168	0.0286959
Cyth3	0.30713144	0.00115203
Cytl1	1.15112133	0.09768936
D2hgdh	-0.4921423	0.08886018
Daam2	0.60444027	0.0146344
Dapk2	0.60651045	0.01813664
Dbil5	0.3841202	0.06463954
Dbt	0.30768453	0.01745855
Ddb2	-0.2787646	0.07002721
Defb27	0.7268058	0.05593551
Dgkb	-0.7197129	0.05669114
Diaph1	0.26426971	0.09137969
Dkk3	-0.9445473	0.0951795
Dlgap1	0.64338312	0.03099922
Dlk1	2.61247481	0.00140687
Dnajb4	0.48029447	0.09308813
Dnph1	-0.1247177	0.03540781
Doc2g	-1.0164758	0.01866169
Dpep1	1.21506911	0.05716717

Dpf1	0.62841176	0.09912888
Dpp4	0.74978893	0.02463332
Dpp7	-0.4426187	0.06459121
Drap1	0.99676054	0.0262608
Duoxa1	-0.308522	0.0262608
Duxbl1	-0.4425872	0.09473484
Ebpl	-0.342941	0.06459121
Edn1	-0.4545426	0.09768936
Eef2k	0.55382924	0.00785664
Efhd2	0.87129846	0.02531006
Egfl7	0.2134844	0.07055603
Eif1	0.1836643	0.09256564
Eif4ebp2	0.56075366	0.03662697
Eif6	0.32131098	0.04346164
Elovl4	0.67467838	0.07278171
Endou	-0.4956212	0.07055603
Eno1	0.52315994	0.0906287
Eno3	-0.788094	0.02581162
Enpp1	0.39689528	0.04842385
Epha3	-1.1806532	0.09457992
Ephb3	-0.7656843	0.0973167
Ephx2	-0.2903014	0.06785276
Eps8	0.35191066	0.08619598
Ero1l	0.71240562	0.09178405
Errfi1	1.18431299	0.01932582
Eva1a	-0.2556371	0.05167644
Fabp4	1.20578384	0.08759111
Fads6	-0.4788755	0.03644476
Fam110c	0.44383333	0.01857659
Fam167a	-0.5375726	0.03662697
Fam19a2	-0.5315403	0.0281468
Fam19a5	0.61806137	0.02059932
Fam213a	1.01114023	0.08588062
Fam213b	-1.2967589	0.07113158
Fam214b	0.43891404	0.04233251
Fam219a	0.42010696	0.00785664
Fam46b	1.15166466	0.00293291
Fam69b	0.19095075	0.00360105
Fas	-0.3545565	0.00554153
Fbxo17	0.42973945	0.09308813
Fbxo32	0.42098944	0.00621992
Fcgbp	0.32326928	0.09308813
Fem1b	0.25540089	0.07808257
Fez2	0.35238357	0.03540781
Fhad1	2.43931772	0.08290051
Fibin	0.48972481	0.08903586
Figf	1.06999432	0.09768936
Fkbp10	0.16401388	0.0262514
Fkbp5	2.04362003	0.01842477
Flnb	-0.2622733	0.01866169
Flt1	0.89125149	0.0262608
Fmo1	0.22315213	0.09321958
Fmo2	1.11229276	0.09216763
Fmo3	1.49716995	0.08619598
Fntb	0.74272111	0.00621992
Foxo1	0.37020195	0.00339241
Fst	0.31962329	0.00410302
Fstl3	1.67790079	0.07421535
Fxyd3	-0.7239312	0.01521807
Gabarapl1	0.70230891	0.08751081
Gal	2.86996572	0.06764533

Galns	-0.1671488	0.00248599
Galnt16	1.56617069	0.04075892
Gatm	-0.6495589	0.04437434
Gbp2	-0.6434447	0.07071726
Gbp5	-0.5063384	0.03607622
Gcnt1	0.63703501	0.07278171
Gdpd5	-0.4136963	0.07552216
Gfra2	0.55149196	0.04085364
Gipc3	0.29941052	0.06039636
Gng10	-0.3829387	0.04612819
Gng13	-0.7940598	0.05915674
Gpd1	1.0459562	0.02681474
Gpm6a	-0.5771644	0.02303985
Gprc5d	-0.4192036	0.07139131
Gpsm1	0.29414155	0.03386707
Gramd3	0.30921617	0.00629748
Gsn	0.60229985	0.06107367
Gstk1	0.34533119	0.09150535
Gstm2	0.35986376	0.06560473
Gucy1a3	-0.2289145	0.09255713
H3f3c	-0.5532046	0.05293884
Hcn4	-0.6752393	0.02049429
Hdac11	-0.7535057	0.09988491
Hdc	-1.0481524	0.04085364
Hexa	-0.2726881	0.0906287
Hgs	0.13362027	0.021638
Hibch	-0.3295345	0.08219625
Hif3a	1.92066248	0.0277816
Hist1h2bcl1	-0.5215082	0.04075892
Hmcn1	-0.4026273	0.03607622
Hmox1	1.16412748	0.033947
Homer3	0.36129183	0.04735717
Hpx	-0.307749	0.07520931
Hrct1	1.04358881	0.01061964
Hsd11b1	1.00191474	0.02266576
Hspa1l	0.31966864	0.05706293
Hspb1	0.27275884	0.04272004
Hspb8	0.26089581	0.09891441
Hvcn1	-0.3886494	0.0262608
Idua	-0.3992185	0.06459121
Ifi27l2b	-0.229705	0.03847172
Ifitm1	-0.1304045	0.05197438
Ifitm2	0.30092393	0.09255713
Igf1r	0.18914509	0.00410302
Igfbp4	-0.7158375	0.09672219
Igfbp6	1.24108475	0.0657096
Il13ra2	-0.5421583	0.07055603
Il18bp	-0.4727055	0.05159012
Iqgap1	-0.1910111	0.04112849
Irf9	0.63285156	0.03701447
Irgm	-0.3530131	0.06459121
Irs2	0.42619087	0.09457992
Itga6	-0.3012891	0.01049748
Ivns1abp	0.9453248	0.07029906
Josd2	0.38404691	0.09081172
Kbtbd3	-0.3394841	0.04075892
Kcnk1	0.6573235	0.07573842
Kif11	-0.5846746	0.05203888
Klf15	0.82395442	0.05167644
Klf9	0.60642874	9.39E-05
Klhl7	-0.2818253	0.06039636

Klhl8	-0.5929334	0.08895145
Krba1	-0.4070942	0.03099922
Krt23	0.5943264	0.03984483
Lama5	1.32690405	0.05915674
Lamc1	0.43138676	0.05810813
Larp6	0.69300216	0.00160926
Ldhd	-0.3260933	0.03395709
Leprel1	0.67260914	0.04112849
Lhfpl2	-0.4826884	0.06930225
Litaf	0.23456196	0.03890191
Lmod1	1.02344659	0.02860476
LOC100365386	0.51557873	0.01932582
LOC680579	0.26450399	0.09308813
LOC684173	-0.2111879	0.01866169
LOC685067	-0.393068	0.0286959
LOC685221	-1.1988534	0.00100253
LOC685431	0.55396772	0.06107367
LOC688286	-0.5272369	0.04114013
LOC688981	-0.6112902	0.0906287
LOC690171	0.06374981	0.06459121
LOC691083	-0.8587362	0.04550058
Lonp1	0.43205278	0.05839056
Lox	0.83912073	0.01219866
Loxl3	0.52095824	0.09228086
Lpar3	0.67156808	0.09308813
Lrrc17	-0.2990809	0.09523328
Lrrc20	0.43008402	0.09457992
Lrrc32	0.86786294	0.04527527
Lrrc59	0.22550804	0.0657096
Lrrn4	-0.3700568	0.0915533
Luc7l3	-0.2785003	0.05840574
Lxn	-0.7196799	0.0591079
Ly96	-0.2446905	0.03984483
Lypd1	-0.8007628	0.02820972
Maff	0.96851821	0.06369604
Maged2	-0.5365901	0.05840574
Manba	-0.5656017	0.08886018
Map1a	0.18644801	0.04258825
Map2k1	0.6399817	0.05760186
Map3k6	0.59946016	0.00084606
Matn2	-0.5938429	0.00544256
Mbnl2	0.40763428	0.09255713
Medag	0.59921909	0.00629748
Mef2bnb	0.52753809	0.03567721
Mettl20	0.9668441	0.04550058
Mfge8	0.48472639	0.05347064
Mfsd2a	0.84875981	0.09178405
Mgll	-0.6274741	0.09172159
Mgst1	1.00616592	6.82E-05
Mlf1	-1.0829425	0.07177971
MLIP	-0.5399097	0.01932582
Mlxipl	1.23687504	0.0146344
Mmp24	1.17915097	0.07553044
Mrap	1.02376951	0.05023565
Msrb2	-0.5765729	0.0262608
Mt-co2	-0.5724328	0.04114013
Mt1	1.56421074	0.0657096
Mt1m	1.55239973	0.02887767
Mt2A	1.4094405	0.02003096
Mtus1	0.56726582	0.080462
Mtx2	-0.2000886	0.06873783

Mx2	-0.7488026	0.09457992
Mybpc2	-0.7230782	0.04075892
Mycn	-0.4500463	0.09216763
Myh7	0.60603723	0.0915533
Mylpf	-0.6323619	0.06147456
Myo6	0.45346693	0.05225571
Mzt1	-0.4955884	0.07109775
N4bp2l1	0.86301999	0.03540781
Nacad	0.54922377	0.0948496
Nbeal2	0.67045878	0.00785664
Nckap5	-0.3434568	0.0052712
Ndel1	0.28581261	0.0286959
Ndn	-0.1429207	0.04075892
Ndrg1	0.87149625	0.05669114
Ndrg4	0.55009157	0.08076176
Nek3	-0.2672855	0.06630559
Nhsl2	0.45283295	0.04279455
Nme3	-0.5295827	0.00544256
Nme4	-0.5865939	0.09308813
Nmnat1	-0.105155	0.0657096
Nnmt	-0.1950854	0.02699214
Notch4	0.83860214	0.04875417
Nov	1.50590434	0.05760186
Npdc1	0.20108338	0.07002721
Npm3	-0.2950575	0.04294003
Nqo1	0.63049116	0.06913226
Nr2c2ap	0.34669144	0.04842385
Nr3c1	-0.5019924	0.09178405
Nr3c2	-0.4874549	0.02091415
Nrep	-1.1872755	0.01521807
Nrip3	0.50189125	0.0886247
Nsun2	0.13274202	0.00785664
Nt5dc3	0.57792225	0.02482424
Ntn4	-0.8300558	0.07109775
Nuak2	-0.8248038	0.07910851
Nucb2	0.76922312	5.17E-05
Numbl	0.78577548	0.01563932
Oas1a	-0.2338847	0.02860476
Oas1b	-0.4260588	0.06647063
Oas1k	0.48385356	0.01524292
Ogdhl	0.07991486	0.02003096
Orm1	5.18715944	0.00544256
Osgin1	0.96236294	0.08229136
P4ha1	0.54264264	0.02916422
P4ha2	0.94249693	0.03847172
Pa2g4	0.1358216	0.09457992
Pacsin3	-0.1930577	0.08132364
Paip2b	0.33016915	0.01932582
Pam	0.51985502	0.04752153
Paqr4	0.60630963	0.00745687
Pbdc1	0.13555133	0.00738241
Pc	0.37992442	0.05447078
Pcbd1	-1.0296897	0.00544256
Pcdh20	1.15381966	0.01866169
Pcolce	-0.2994942	0.06993342
Pdap1	0.18974977	0.01521807
Pdcd6	0.33631648	0.09294903
Pde1a	-0.4394828	0.01596592
Pdia2	-0.7053527	0.09268812
Pdia4	-0.3504004	0.09963191
Pdk4	0.53319663	0.06459121

Pdlim2	-0.5159241	0.02802798
Pfkfb3	1.74665004	0.0262608
Pfkl	0.96399217	0.04320849
Pfkp	0.78356976	0.08076176
Pgam1	0.46651068	0.02172718
Pgf	1.24697731	0.01857494
Phkb	-0.5080579	0.03540781
Phtf2	-0.5867595	0.06993342
Phyhd1	1.12853253	0.0229974
Pim3	0.45235998	0.01563932
Pir	0.84731313	0.02820972
Pla1a	0.72287191	0.05940213
Pla2g4b	0.35581892	0.0103718
Plau	-0.5218942	0.03540781
Plin4	1.26885856	0.0915533
Pmm2	0.23926075	0.01521807
Pnma2	-0.6737606	0.09544734
Pnpo	0.32630739	0.0015542
Podxl	1.31682142	0.03448129
Podxl2	-0.7903229	0.05347064
Polr1c	0.13282043	0.04780599
Por	0.36954516	0.02482424
Ppap2c	0.13880568	0.03156601
Pparg	0.48801649	0.04505752
Ppfia4	-0.5327231	0.08670538
Ppp2r1b	-0.3303377	0.08076176
Ppp2r3a	-0.5165791	0.09172159
Praf2	0.43338859	0.0973167
Prg4	1.13033027	0.07760099
Prima1	0.5962723	0.0146344
Prmt5	0.18726247	0.06647063
Prodh	1.13279926	0.01928903
Prosc	0.44627073	0.06459656
Prr13	0.36759686	0.08004508
Prrx2	-0.4925374	0.08631645
Ptgfr	-0.8579439	0.05824917
Ptplb	1.42075302	0.03448129
Ptpn11	0.64595319	0.05593551
Pus7	0.18087237	0.07587523
Pvalb	-0.1653603	0.0973167
Pyroxd2	-0.6407433	0.09755694
Qsox1	1.11286985	0.04279455
Rab13	-0.375736	0.05840574
Rab32	-0.3989632	0.05589522
Ralgps2	-0.568518	0.07808257
Ramp1	1.2951413	0.04780599
Ranbp3l	-0.2979346	0.0004827
Rapgef4	0.61889865	0.04842385
Rapgef5	1.17850732	0.00160926
Rapsn	-0.8427456	0.0841198
Rasgrp1	0.74620401	0.00410302
Rbm3	-0.5132631	0.04527527
Rec114	-0.750849	0.04823426
Reep5	0.04548833	0.00360105
Relt	0.4605687	0.03984483
Retsat	0.64226278	0.08575298
Rexo2	0.4345996	0.06691975
Rfc3	-0.5902951	0.0841198
Rgcc	1.84160342	0.04258825
RGD1304982	-0.6424642	0.09768936
RGD1307315	-0.184945	0.09891441

RGD1561149	0.59372899	0.05593551
RGD1561161	-0.5154617	0.07777064
RGD1561849	-0.6569517	0.0064057
RGD1562055	0.76996488	0.02882862
RGD1562658	-0.3358604	0.07400661
Rgs12	-0.5782504	0.09159358
Rgs3	-0.4746431	0.0674962
Rhobtb1	1.13440672	0.01521807
Rhoj	1.13684502	0.03340679
Rin3	0.92842644	0.09768936
Ripk3	-0.1787938	0.00629748
Rn50_1_1736.2	0.54015474	0.08346069
Rn50_X_0691.2	-0.3546306	0.03721574
Rnase3	0.46124098	0.0064057
Rnase4	0.42545127	0.06317843
Rnf170	-0.818347	0.06295789
Rpl26-ps1	0.45771706	0.00896349
Rprml	1.02006075	0.0319692
Rps26	0.38370738	0.04505752
Rrp8	0.32264485	0.03964043
RT1-T24-4	-0.1289982	0.06531145
Rwdd3	-0.588114	0.09172159
S100a10	0.48882614	0.03099922
S100a6	0.51513825	0.06764533
Samd11	-0.8790902	0.0906287
Sbk1	-0.5651376	0.09294903
Scgb1a1	1.10547349	0.03099922
Scimp	-0.4328182	0.0978117
Scnn1a	1.00900013	0.06148794
Sdc1	-0.4471389	0.03536404
Sdcbp2	0.96737713	0.06338434
Selplg	0.04149111	0.03099922
Serf1	-0.5170086	0.01763871
Sergef	0.26685311	0.06691975
Serpina3n	1.65943915	0.0131736
Serpinb9	-0.7087935	0.09151955
Serpine1	0.92158455	0.06459121
Serping1	0.36490262	0.05840574
Sfrp2	-1.1010296	0.05365168
Sfxn3	0.08177001	0.09255713
Sgcb	-0.3462826	0.06560473
Sh3bgrl	-0.7172042	0.09547937
Sh3kbp1	-0.2578268	0.01763871
Sh3pxd2b	0.56495553	0.09916406
Ska2	-0.5738881	0.00410302
Slamf8	-0.4133409	0.05329896
Slc22a1	0.55381005	0.08391774
Slc24a4	0.38339658	0.07861867
Slc25a4	0.50190259	0.06148794
Slc29a1	-0.5716646	0.05210007
Slc2a1	0.58994002	0.05365168
Slc2a13	0.4610171	0.01625268
Slc30a4	-0.3040259	0.0462988
Slc35d2	-0.484268	0.09457992
Slc37a1	-0.4495636	0.09672219
Slc43a3	0.83543168	0.00629748
Slit3	0.57233205	0.05167644
Slitrk5	0.19659638	0.09172159
Smim4	0.58343483	0.01521807
Smoc1	-0.6177311	0.04112849
Smoc2	-0.8479239	0.08989671

Smpdl3b	0.10494179	0.00745687
Snn	-0.7535707	0.06011422
Snrpa	-0.3645848	0.02820972
Sorbs1	0.82948245	0.0146344
Sorcs1	1.90510319	0.09530694
Sorcs2	-0.8211798	0.01563932
Sorl1	-0.5384696	0.04219271
Sox8	0.73955438	0.0262608
Spata13	0.8736878	0.02860476
Spef1	0.60346778	0.0022271
Sphkap	-1.1316162	0.07055603
Spire1	0.14820754	0.06912427
Spsb4	0.28963294	0.0906287
Sqstm1	0.64374451	0.04075892
Src	-0.5620882	0.09172159
Srpk3	-0.6234592	0.00482803
Srsf5	-0.2101401	0.06647063
St3gal2	0.38222158	0.09988491
Stac2	0.40033302	0.08814355
Stard10	-0.5180749	0.00139495
Stat1	-0.5753592	0.09255713
Stbd1	0.16863724	0.02003096
Steap2	0.72675328	0.05669114
Stk39	-0.2388032	0.0262608
Sult1a1	3.05358355	0.01049748
Syne3	0.2681782	0.01482371
Tacc1	0.72219139	0.05764797
Tacc2	0.23518913	0.08901373
Tacc3	-0.7490697	0.07113158
Taf9b	-0.5702571	0.00985657
Tcaim	-0.4552217	0.05760186
Tceal1	-0.6282616	0.08670538
Tec	-0.4519516	0.06459121
Tenc1	0.55857092	0.06459121
Tesc	0.81252346	0.08227884
Thbd	0.84570493	0.03271195
Thop1	0.28643508	0.01063713
Timp4	2.25645724	0.04476055
Tinagl1	0.74730799	0.00293291
Tmem115	0.14052016	0.01061964
Tmem182	-0.5317628	0.06691975
Tmem43	0.24117534	0.06369604
Tmem71	-0.9403921	0.09457992
Tmsb10	-0.1463424	0.04189714
Tnfrsf11b	-0.622941	0.04346164
Tnfrsf17	2.58467711	0.01521807
Tnfrsf1a	0.23435141	0.09178405
Tns1	0.64217443	0.01219866
Trappc2	-0.7330193	0.03706967
Triqk	-0.7298085	0.09457992
Tspan13	-0.6445736	0.07005168
Tspan17	0.39473507	0.0146344
Tspan6	-0.5761043	0.05664271
Tubb2b	-0.503716	0.08219625
Tubb3	-0.455773	0.01211599
Txnrd1	0.51448981	0.07634359
Uap1	0.58184347	0.09916406
Ube4b	0.23674498	0.02989391
Ubxn6	0.09035839	0.0997824
Uck2	0.53120192	0.0973167
Ucma	0.69915278	0.00544256

Ugt1a3	0.60108301	0.03026291
Usp18	-0.7043701	0.05664271
Veph1	-0.883118	0.08979751
Wbp1	-0.5732877	0.08670538
Wnk2	0.81871926	0.02857668
Wnt2b	0.34050514	0.08355126
Wscd1	0.64774036	0.06300925
Xdh	0.96767814	0.09151955
Ybx2	-0.3372529	0.01857494
Ypel1	-0.6885099	0.07055603
Zfp189	0.90812434	0.03662697
Zfp365	-0.3282007	0.05776523
Zfp386	-0.418498	0.08597666
Zfp414	-0.5244623	0.04333341
Zfp763	-0.1997969	0.09137969
Zfp955a	-0.2231959	0.08076176
Zscan2	-0.2218853	0.02219748
Table S2. Differentially expressed transcripts. List of all differentially expressed transcripts. NF vs DCM q<0.10.		

miRNA	Fold Change	qValue
hsa-miR-126-3p	-0.79	0.000621
hsa-miR-574-3p	-0.96	0.000621
hsa-miR-628-5p	-0.83	0.001505
hsa-miR-140-3p	-0.59	0.001932
hsa-miR-193b-3p	-0.81	0.001932
hsa-miR-200c-3p	-0.97	0.001932
hsa-miR-342-3p	-0.84	0.001932
hsa-miR-454-3p	-0.83	0.001932
hsa-miR-195-5p	-0.61	0.002365
hsa-miR-374b-5p	-0.79	0.002365
hsa-miR-133a-3p	-1.16	0.002591
hsa-miR-139-5p	-0.81	0.002591
hsa-miR-155-5p	-0.86	0.002591
hsa-miR-484	-0.49	0.002591
hsa-miR-125b-5p	-1.02	0.002703
hsa-miR-146b-5p	-0.72	0.002703
hsa-miR-150-5p	-0.78	0.002703
hsa-miR-26a-5p	-0.76	0.002703
hsa-miR-374a-5p	-0.74	0.002703
hsa-miR-375	-0.64	0.002703
hsa-let-7c-5p	-0.88	0.003010
hsa-miR-203a-3p	-0.95	0.003010
hsa-miR-28-3p	-0.73	0.003644
hsa-miR-331-3p	-0.75	0.003913
hsa-miR-191-5p	-0.59	0.005243
hsa-miR-214-3p	-0.89	0.005828
hsa-miR-365a-3p	-0.73	0.005828
hsa-miR-142-5p	-0.71	0.005869
hsa-miR-186-5p	-0.47	0.006923
hsa-miR-215-5p	-0.64	0.007398
RNU48-001006	-1.04	0.007441
hsa-miR-122-5p	-0.71	0.007742
hsa-miR-31-5p	-0.75	0.008142
hsa-miR-339-3p	-0.55	0.008451
hsa-miR-125a-5p	-1.16	0.008543
hsa-miR-197-3p	-0.62	0.008543
hsa-miR-30b-5p	-0.69	0.009241
hsa-miR-145-5p	-1.01	0.010135
hsa-miR-532-3p	-0.69	0.010135
hsa-miR-140-5p	-0.60	0.010135
hsa-miR-26b-5p	-0.53	0.010741
hsa-miR-29a-3p	-0.58	0.011100
hsa-miR-30c-5p	-0.66	0.011100
hsa-miR-99b-5p	-0.70	0.011100
hsa-let-7b-5p	-0.54	0.011348
hsa-miR-24-3p	-0.57	0.011348
hsa-miR-204-5p	-0.62	0.013905
hsa-miR-363-3p	-0.72	0.013905

hsa-miR-885-5p	-0.57	0.014769
hsa-miR-27b-3p	-0.70	0.016100
hsa-miR-744-5p	-0.77	0.016100
hsa-miR-192-5p	-0.47	0.016342
hsa-miR-29c-3p	-0.54	0.017303
hsa-miR-340-5p	-0.54	0.017303
hsa-miR-345-5p	-0.53	0.018170
hsa-miR-106b-5p	-0.54	0.021001
hsa-miR-139-3p	-0.52	0.021001
hsa-miR-146a-5p	-0.52	0.021001
hsa-miR-489	-0.86	0.021501
hsa-miR-376c-3p	-0.70	0.024166
hsa-miR-485-3p	-0.62	0.025819
hsa-miR-27a-3p	-0.63	0.029193
hsa-miR-301a-3p	-0.54	0.029866
hsa-miR-212-3p	-0.59	0.029952
hsa-miR-19a-3p	-0.49	0.030078
hsa-miR-101-3p	-0.36	0.030964
hsa-miR-106a-5p	-0.40	0.030964
hsa-miR-451a	-0.40	0.032936
hsa-miR-17-5p	-0.41	0.035094
hsa-miR-218-5p	-0.62	0.037960
hsa-miR-199a-3p	-0.62	0.040511
hsa-let-7g-5p	-0.56	0.042882
hsa-miR-152-3p	-0.56	0.044935
hsa-miR-181a-5p	-0.54	0.051109
hsa-miR-370-3p	-0.70	0.062168
hsa-let-7d-5p	-0.49	0.068553
hsa-miR-19b-3p	-0.40	0.068553
hsa-miR-142-3p	-0.57	0.071907
hsa-miR-590-5p	-0.31	0.075760
hsa-miR-28-5p	-0.49	0.077713
hsa-miR-143-3p	-0.76	0.078028
hsa-miR-376a-3p	-0.62	0.081457
hsa-miR-132-3p	-0.39	0.090771
hsa-miR-148a-3p	-0.33	0.092245

Table S3. Circulating miRNA significantly dysregulated in serum of dilated cardiomyopathy patients. List of all dysregulated circulating miRNA, NF vs DCM, $q<0.1$.

gene ID	foldChange	qValue
ADIPOQ	1.27092046	0.018028316
ADRBK1	1.788058247	0.091211557
ANGPT2	1.559455133	0.073343204
ATP5B	0.714160164	0.038874434
B2M	0.778322309	0.019111631
BIRC3	-0.553523315	0.051999697
BIRC7	-0.379006161	0.08434117
BMP10	0.934036756	0.08490911
BSG	0.876320434	0.100416428
BTK	1.06966451	0.036070374
C1QA C1QB C1QC	-0.297414996	0.035196173
C1QBP	-0.325216639	0.064243597
CAMK2B	1.188261423	0.030115571
CAMK2D	1.325478635	0.036070374
CCL15	0.790947878	0.102124066
CCL24	-0.221323407	0.074296923
CCL28	2.254752799	0.081006574
CD27	-0.359782824	0.109815652
CD55	-0.698278889	0.046404195
CD84	0.406812097	0.082184505
CDH3	-0.627197505	0.065572096
CHL1	-0.733858928	0.035196173
CKM	-0.960059195	0.106846199
CNDP1	-2.421556115	0.08490911
CNTF	-0.52549833	0.035736715
CRLF1 CLCF1	-0.428275441	0.08490911
CSK	1.827753075	0.017344244
CST3	0.724446078	0.017344244
CTSH	0.520944839	0.104439691
CXCL16	0.572059058	0.018028316
DCN	0.551190809	0.109970496
DSC3	-0.847729715	0.064590854
ECE1	-0.830073535	0.019111631
EFNB3	0.600832391	0.08490911
EGFR	-0.723545585	0.068025685
FCGR1A	1.109285342	0.017344244
FYN	0.788221338	0.054642224
GFRA3	0.609537305	0.068025685
GPNMB	-0.521334029	0.036070374
GPT	0.507794165	0.091633836
GSK3A GSK3B	1.143228748	0.038874434
GSN	-0.608350833	0.08490911

HCE000414	-0.0237112	0.08490911
HDGFRP2	0.570327096	0.055032766
HFE2	-1.03470188	0.036070374
HMGB1	1.439235764	0.019111631
HNRNPA2B1	1.364808981	0.08434117
HNRNPK	-0.64876325	0.017544144
HSP90AA1 HSP90AB1	0.837641837	0.068665868
IGFBP2	1.185422833	0.019111631
IGFBP4	0.426670233	0.08434117
IL27 EBI3	-0.338408827	0.019111631
IL4R	-0.614172005	0.091211557
ITGA1 ITGB1	1.494840254	0.038874434
KIR2DL4	0.867711798	0.056947569
KIT	-0.666241932	0.065572096
L1CAM	-0.980346925	0.046404195
LCMT1	0.631101083	0.035736715
LGALS8	-0.496033544	0.023110909
LYN	1.641577514	0.035196173
MAPK3	1.208736523	0.018028316
MAPKAPK2	1.150888562	0.023110909
MDK	4.514149307	0.003357918
MET	-0.637750293	0.08490911
METAP1	1.193940983	0.023347982
MMP7	1.257530836	0.104439691
NID1	0.780850377	0.051428825
NID2	0.572683679	0.081006574
NLGN4X	0.87253887	0.071138015
NME1	1.268462881	0.01919371
NPPA	1.682920501	0.041119692
NR1D1	-0.551275875	0.089082417
NRXN3	-0.557124334	0.08434117
NTF4	-0.297877877	0.081006574
NTRK2	-0.840937636	0.038874434
PDE5A	1.531194332	0.035196173
PKM2	1.299040937	0.030115571
PON1	-0.266239094	0.050350976
PPID	1.433626852	0.081006574
PRDX5	-0.565316037	0.019111631
PRKAA1 PRKAB1 PRKAG1	-0.629809131	0.06952404
PRKCB	1.665598443	0.019111631
PTPN6	1.637574492	0.017803948
REN	3.185742741	0.030792025
RET	-1.436245306	0.035736715

RGMB	-0.574369214	0.020825427
ROR1	-0.771578279	0.071278018
SEMA3E	-1.439362103	0.019111631
SERPINA3	0.490810666	0.036745527
SFRP1	2.191540139	0.055821011
SFTPD	2.12223774	0.019111631
SIGLEC9	4.039367327	0.04363951
SIRT2	1.796669539	0.046404195
SNX4	1.388778635	0.020825427
SPHK2	-0.369068291	0.089082417
SPINT2	0.616017707	0.091211557
SPOCK2	0.948116109	0.03541443
SRC	2.093889513	0.020825427
STK17B	0.979591265	0.023379742
TFF3	0.97909478	0.017356683
THBS2	2.394766134	0.023110909
TNFRSF12A	-0.425592501	0.036070374
TNFRSF18	-0.441602515	0.051079859
TNNI3	1.809861892	0.08434117
TPSB2	-1.564301883	0.019111631
TPT1	1.158975637	0.038874434
VWF	1.322736979	0.08490911
WNT7A	-0.460412364	0.081006574
YWHAB,YWHAE,YWHAG,YWHAH,YWHAQ,YWHAZ,SFN	1.089448844	0.035736715

Table S4: Differentially expressed protein. List of all differentially expressed protein, NF vs DCM, $q<0.10$.

Top Canonical Pathways: miRPathDB
**Canonical Pathways (all p<0.05 by Benjamini-Hochberg adjustment)**
miRNAs in cancer
Pathways in cancer
Cell cycle
p53 signaling pathway
Focal adhesion
PI3k AKT signaling pathway
Bladder cancer
HTLV infection
Non small cell lung cancer
Glioma
Melanoma
Hepatitis B
Colorectal cancer
Pancreatic cancer
Prostate cancer
Proteoglycans in cancer
Chronic myeloid leukemia
FoxO signaling pathway

Table 5. Circulating miRNAs are dysregulated in pediatric DCM patient serum, which is associated with changes in canonical pathway. Significantly dysregulated canonical pathways from serum DCM patient miRNAs identified using miRPathDB.
